# Phase Ib trial of inhaled iloprost for the prevention of lung cancer with predictive and response biomarker assessment

**DOI:** 10.3389/fonc.2023.1204726

**Published:** 2023-08-30

**Authors:** York E. Miller, Moumita Ghosh, Daniel T. Merrick, Brandi Kubala, Eva Szabo, Lisa Bengtson, Masha Kocherginsky, Irene B. Helenowski, Kelly Benante, Tia Schering, Jihye Kim, Hyunmin Kim, Duc Ha, Raymond C. Bergan, Seema A. Khan, Robert L. Keith

**Affiliations:** ^1^Division of Pulmonary Sciences and Critical Care Medicine, University of Colorado, Aurora, CO, United States; ^2^Pulmonary and Critical Care Section, RMR VAMC Rocky Mountain Regional Veteran Administration Medical Center, Aurora, CO, United States; ^3^Department of Pathology, University of Colorado, Aurora, CO, United States; ^4^Cancer Center Clinical Trial Core, University of Colorado, Aurora, CO, United States; ^5^Division of Cancer Prevention, National Cancer Institute, Bethesda, MD, United States; ^6^Department of Preventative Medicine, Northwestern University, Evanston, IL, United States; ^7^Robert H. Lurie Comprehensive Cancer Center, Northwestern University, Evanston, IL, United States; ^8^Department of Quantitative Health Sciences, Cleveland Clinic, Cleveland, OH, United States; ^9^Department of Genetics and Genome Sciences, Case Western Reserve University, Cleveland, OH, United States; ^10^Fred and Pamela Buffett Cancer Center, Division of Oncology & Hematology, Genitourinary Oncology, University of Nebraska, Evanston, IL, United States; ^11^Department of Surgery, Northwestern University, Omaha, NE, United States

**Keywords:** lung squamous cell cancer, bronchial dysplasia, iloprost, medical prevention, epithelial progenitors

## Abstract

**Introduction:**

Iloprost, a prostacyclin analog, has lung cancerpreventive activity in preclinical models and improved dysplasia in former smokers in a phase IIb trial. Oral iloprost is currently unavailable. We performed a phase Ib trial of inhaled iloprost in former smokers to assess tolerance and compliance.

**Methods:**

Participants self-administered nebulized iloprost (5ug) or placebo four (QID) or two (BID) times daily. As QID dose was well tolerated and due to expiration of the placebo, the BID dosing and placebo were eliminated early on in the trial. Bronchoscopy with biopsyat six standard sites was performed at treatment initiation and two months post-iloprost, with exploratory histological analysis. Bulk RNA sequencing, single cell RNA sequencing and an in vitro assay of epithelial progenitor cell iloprost response were performed on a subset of biopsies in an exploratory investigation of response mechanisms and predictive biomarkers.

**Results and discussion:**

Thirty-four of a planned 48 participants were recruited to the trial.Inhaled iloprost was well tolerated with no adverse events > grade 2. Compliance was 67% in the QID group. The trial was not powered to detect histologic response and none was found. Bulk RNA sequencing of biopsies pre/post iloprost suggest that iloprost is immunomodulatory and downregulates cell proliferation pathways. Single cell RNA sequencing showed an increase in CD8-positive T cells with upregulation of genes in interferon γ signaling. In vitro iloprost response by epithelial progenitor cells correlated with histologic response with kappa coefficient of 0.81 (95% CI 0.47, 1.0). Inhaled iloprost was well tolerated with suboptimal compliance. Molecular analysis suggested that iloprosthas immunomodulatory and antiproliferative effects.The progenitor cell iloprost response assay may be a promising avenue to develop predictive biomarkers.

**Clinical trial registration:**

https://clinicaltrials.gov/study/NCT02237183, identifier NCT02237183.

## Introduction

Lung cancer is the most common cause of cancer death in men and women in both the United States and the world (1). The most effective preventive measures are the primary prevention of smoking and smoking cessation (2). Most lung cancer cases in the United States are diagnosed in former smokers and further interventions beyond smoking cessation are needed.

Prostacyclin and analogs decrease endothelial tumor cell adhesion, preventing experimental metastasis and inhibiting the growth of established micrometastases in preclinical studies (3). Well-described activities of prostacyclin include platelet inhibition, vasodilation, and suppression of inflammation. Surfactant protein C (SPC) promoter-directed overexpression of prostacyclin synthase (PGIS) in mice prevents the development of pulmonary adenomas in several chemical carcinogenesis models, including urethane, 3-methyl cholanthrene/butylated hydroxytoluene, and tobacco smoke (4, 5).

Investigations have been hindered by the short half-life of prostacyclin and its analogs (3). Iloprost is a prostacyclin analog with improved stability and is used in intravenous and inhaled forms to treat pulmonary hypertension. Oral and inhaled iloprost both prevent pulmonary adenomas in mice (6, 7). A double-blind phase IIb trial of oral iloprost or placebo in individuals at high risk for lung cancer showed improvement in endobronchial dysplasia in former, but not current smokers (8), the only phase IIb trial to have achieved a statistically significant effect on a predetermined dysplasia endpoint (9). Approximately 50% of former smokers responded to oral iloprost, demonstrating the need for a predictive marker to identify responders.

As the inhaled preparation of iloprost is currently approved for pulmonary hypertension and oral iloprost is not available, we designed a phase Ib trial to assess tolerability and compliance of inhaled iloprost in these individuals, with exploratory endpoints including histology and lung function to assess potential for clinical benefit.

## Methods

### Study design

**Figure 1:**
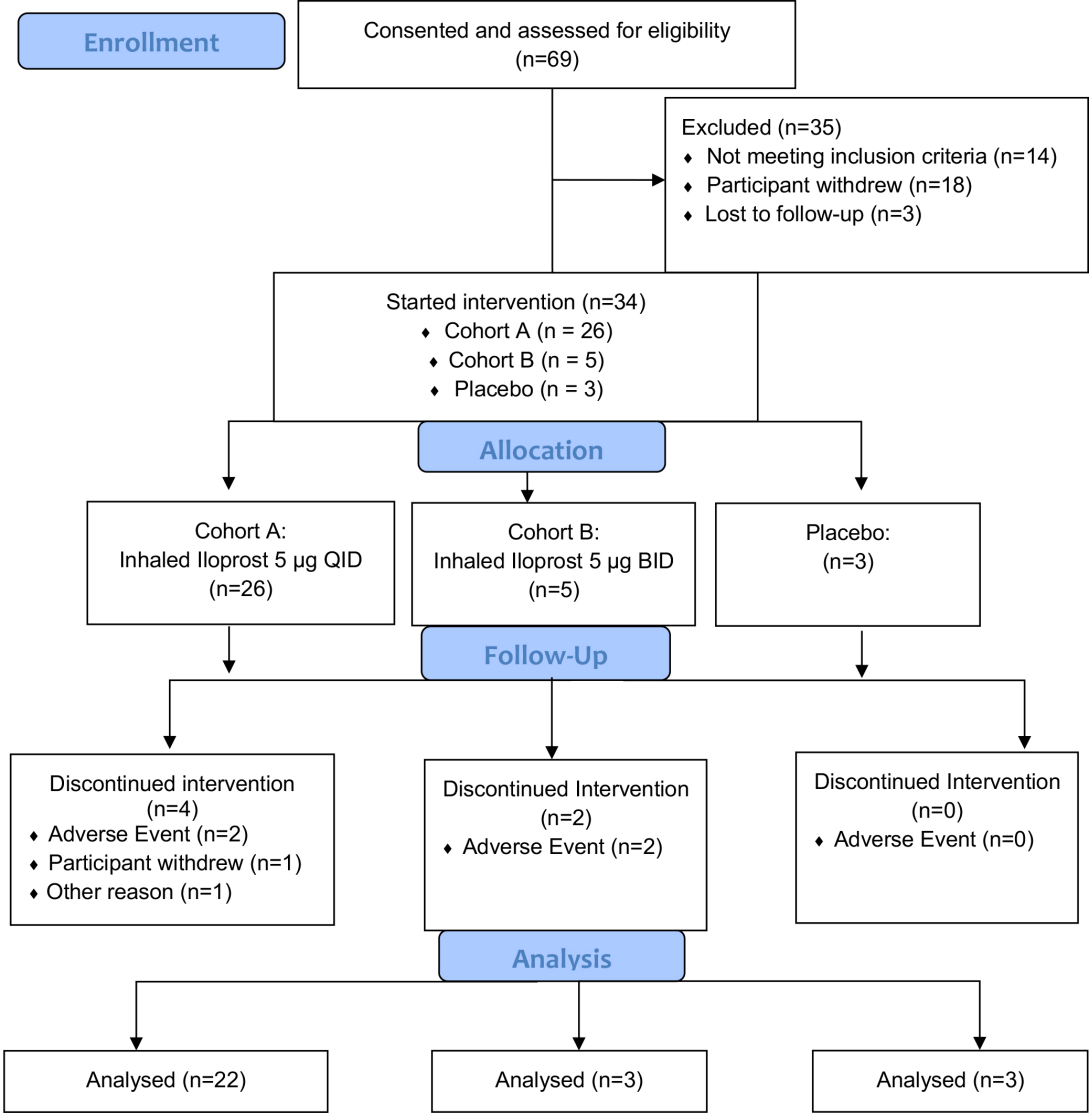
Study design and enrollment in the trial.

The current trial (NCT02237183) was initially designed as a randomized, double-blind, dose de-escalation trial of inhaled iloprost compared to placebo with two cohorts. The standard use of inhaled iloprost for pulmonary hypertension requires eight or more daily treatments; we considered this to be impractical for a prevention application and chose QID and BID treatments as more realistic. Ampules containing 5 µg of iloprost and placebo were made available by the manufacturer, Bayer. Cohort A (*n* = 16) was to self-administer 5 µg of iloprost QID and Cohort B (*n* = 16) was to self-administer 5 µg of iloprost BID, both for 60 days. Placebo was to be self-administered to 8 participants each in the same schedule as in Cohorts A and B, resulting in a total of 48 participants planned. The final trial schema is summarized in **Figure 1**.

Inclusion criteria included a smoking history of at least 20 pack-years with abstinence for >12 months and either sputum cytologic atypia (≥ mild) or a history of endobronchial dysplasia (≥ mild) within the past 12 months; ages 18 to 85 years old; ECOG performance status of ≤1; normal organ function; the ability to safely undergo bronchoscopy; and the ability and willingness to give informed consent. Exclusion criteria included current use of thiazolidinediones; treatment with iloprost at any time; current or prior malignancy within the past 6 months (other than curatively treated non-melanoma skin cancer, cervical carcinoma *in situ*, and bladder carcinoma *in situ*); receipt of chemotherapy or radiotherapy within the previous 6 months; pregnancy or breastfeeding; and the use of anticoagulants other than aspirin or non-steroidal anti-inflammatory drugs. Participants were recruited from the Pulmonary clinics at the Rocky Mountain Regional Veterans Administration Medical Center and by referral from University of Colorado Hospital clinics.

### Screening and testing

After a pre-screening visit, two sequential 3-day sputum samples were collected, one in Saccomanno’s fluid and the other in a similar but proprietary fixative provided by VisionGate, Inc (10). Individuals who produced sputum with ≥ mild atypia were invited to participate in the trial and underwent screening tests, including physical exam, labs, baseline bronchoscopy, full pulmonary function testing (PFTs) including diffusing capacity for carbon monoxide (DLCO), EKG, exhaled carbon monoxide (CO), 6-minute walk test (6MWT), St. George Respiratory Questionnaire (SGRQ), and Chronic Obstructive Pulmonary Disease Assessment Test (CAT) questionnaire administration.

### Agent administration

Iloprost or matched placebo was delivered by the FDA-approved I-neb Adaptive Aerosol Delivery (AAD) device (Accredo). Each participant was taught to self-administer the experimental agent. Treatments last 4–10 min and the device measures compliance and delivered dose. During the initial training visit, a 2.5-µg test dose was administered and participants delivered the full 5-µg doses subsequently. Intolerance was defined as coughing lasting more than 10 min, systolic blood pressure decrease of more than 20 mm Hg, or flushing lasting more than 10 min.

### Bronchoscopy

White light bronchoscopy was performed at trial initiation and after 60 days of treatment while participants were still taking drug or placebo. Six predetermined sites, namely, the carinae of the right upper lobe, right middle lobe, superior segment of the right lower lobe, left upper lobe, left upper division/lingula, and superior segment of the left lower lobe, were biopsied, along with any additional sites that appeared abnormal. Additional biopsies were taken for RNA and progenitor cell culture.

### Trial procedures

Blood and urine were obtained prior to each bronchoscopy, and sputum was collected at trial conclusion. Interval visits were carried out on days 15 (telephone) and 30. Pre-study testing was repeated at the 60-day visit, including PFTs, 6MWT, the SGRQ, and CAT. On day 90, a post-intervention physical exam, history, and toxicity assessment was conducted.

### Histologic analysis

H & E endobronchial biopsies were graded according to the World Health Organization (WHO) grading system (11) and assigned a score according to the following scale: 1 = normal bronchial epithelium; 2 = reserve cell hyperplasia; 3 = squamous dysplasia; 4 = mild dysplasia; 5 = moderate dysplasia; 6 = severe dysplasia; 7 = carcinoma *in situ*; and 8 = invasive carcinoma. All biopsies were graded in a blinded fashion by the study pathologist (DTM). Biopsies were also assigned a visual inflammation score based on estimated cellularity attributed to inflammatory cells, with 0 = no significant inflammation, <5%; 1 = mild, 5%–25%; 2 = moderate, 25%–75%; 3 = severe, >75%.

### Bulk and single-cell RNAseq analysis of bronchial biopsies to investigate effect of iloprost

Bronchial biopsies frozen in OCT were used for bulk mRNAseq studies. Total RNA was purified using an RNA extraction microkit (Qiagen), followed by mRNA library prep (Illumina) and sequencing (NovaSEQ next-gen sequencer). The data analysis pipeline included trimming paired-RNA fastq files and mapping to human genome hg38 using STAR (12). The fragments per exon kilobase per million mapped reads (FPKM) values were calculated using RNAseq by expectation maximization (RSEM) (13). eBayes (limma R package) was used to fit the model that considers participant heterogeneity, batch effect, and histology. Significant differences between groups (pre-iloprost, post-iloprost, and placebo) were calculated using moderated *t*-statistics *(* 14, 15). The FPKM tables of pre-iloprost vs. post-iloprost and pre- and post-placebo were used as input to identify differentially expressed pathways by GSEA (gene set enrichment analysis) (16) considering Hallmark as reference database (17). The effective gene sets (normalized *p* < 0.01) of each pathway were collected and visualized using custom R codes.

For single-cell RNAseq (scRNAseq), biopsies were proteolytically digested to prepare single-cell suspensions and used in the Chromium Single-cell 3’ solution capture system from the 10x Genomics platform. Cell ranger 5.0 was used to create cell by gene count tables, preprocessed using Seurat R package (18) and batch-corrected by harmony in R (19). The Uniform Manifold Approximation and Projection (UMAP) and violin plots were generated by the Seurat R package, and heatmap plots of average expression were produced by using the ComplexHeatmap R package (20).

### Epithelial basal progenitor culture to determine iloprost *in vitro* response

An air–liquid interface (ALI) culture method for bronchial basal progenitor cells following previously described methods was developed (21). Briefly, biopsies were proteolytically digested and single-cell suspensions were used to grow basal progenitor cells following the methods described before (21, 22). Clonal basal cells were then plated on collagen-coated transwell membrane inserts and used for *in vitro* ALI differentiation. Cells were treated with either vehicle or 1 µM iloprost during the entire culture period (6 weeks) with drug replenishment every 48 h. Membranes were fixed with 10% neutral buffered formalin, embedded in paraffin, sectioned, and H & E stained to determine epithelial histology.

### Statistical design and analysis

The primary objective of the trial was to evaluate the toxicity of inhalational iloprost administered QID to participants for 2 months using CTCAE v4.0 and compliance. Exploratory endpoints for which the trial was not powered to assess included effect on endobronchial histology, expectorated sputum cytology, and pulmonary function. Participant characteristics and adverse events are summarized using descriptive statistics. Histology was assessed on the WHO 1–8 scale at each biopsy site at baseline and post-treatment, and changes in worst-grade histology were analyzed using the Wilcoxon signed-rank test due to the discrete nature of the outcome. Changes in PFT, 6MWTs, and SGRQ and CAT scores were also analyzed using the Wilcoxon signed rank test.

### Study design modifications, enrollments, and dropouts

Because of the expiration of the placebo and the inability to procure more, the study design was modified to eliminate the placebo group after three individuals were enrolled. Owing to the resultant delays to study completion and given that the QID treatment schedule was well tolerated, the protocol was amended to eliminate the BID cohort (cohort B) after five individuals were enrolled.

### Data availability

All the de-identified genomic data will be available at the NCBI db-GAP database.

## Results

### Demographics and study description

Screening for potential participants occurred in the Rocky Mountain Regional VAMC pulmonary clinics from 15 November 2015 to 27 November 2019. Of 69 consented individuals, 21 withdrew from the study or were lost to follow-up prior to sputum collection, 14 had screen failures (insufficient sample or lack of cytologic atypia), and 31 met the inclusion criterion of at least mild atypia (18 = mild; 11 = moderate; 2 = severe), as shown in **Figure 1**. All enrolled in the trial and underwent at least one bronchoscopy (**Figure 1**). Three additional individuals were enrolled on the basis of known mild or worse endobronchial dysplasia on prior bronchoscopy. Of the 34 enrolled participants, 26 individuals received iloprost QID, 5 received iloprost BID, and 3 received placebo. The baseline characteristics of the study participants are summarized in **Table 1**. Participants were predominantly male (88%), Caucasian (88%), and elderly (mean age = 64.6; range = 32–84 years old). Median (range) for pack-years smoked was 36 (21.5–88), and years since quit smoking was 19.4 (1–44). Average lung function FEV1% predicted was 86.3% (range = 29%–127%), and FEV1/FVC was 0.72 (0.35–0.92). Of 34 participants, 8 (25%) had COPD by the standard definition of FEV1/FVC < 0.70.

**Table 1:** Demographics and baseline characteristics of study participants.

Characteristic	Overall, *N* = 34	Cohort A Iloprost QID, *N* = 26	Cohort B Iloprost BID, *N* = 5	Placebo, *N* = 3
Age				
Mean (SD)	64.65 (10.46)	64.04 (11.69)	67.20 (4.21)	65.67 (6.11)
Median (Range)	67.00 (32.00, 84.00)	66.50 (32.00, 84.00)	67.00 (61.00, 72.00)	67.00 (59.00, 71.00)
Sex				
Female	4 (12%)	4 (15%)	0 (0%)	0 (0%)
Male	30 (88%)	22 (85%)	5 (100%)	3 (100%)
Race				
Asian	1 (2.9%)	1 (3.8%)	0 (0%)	0 (0%)
Black or African American	2 (5.9%)	1 (3.8%)	1 (20%)	0 (0%)
Native Hawaiian or Other Pacific Islander	1 (2.9%)	1 (3.8%)	0 (0%)	0 (0%)
White	30 (88%)	23 (88%)	4 (80%)	3 (100%)
Ethnicity				
Hispanic or Latino	1 (2.9%)	1 (3.8%)	0 (0%)	0 (0%)
Not Hispanic or Latino	33 (97%)	25 (96%)	5 (100%)	3 (100%)
Smoking Status				
Former Smoker	34 (100%)	26 (100%)	5 (100%)	3 (100%)
Number of years smoked cigarettes				
Mean (SD)	30.15 (10.30)	28.46 (9.45)	38.20 (11.99)	31.33 (12.06)
Median (Range)	30.00 (13.00, 55.00)	28.00 (13.00, 47.00)	34.00 (24.00, 55.00)	30.00 (20.00, 44.00)
Number pack years smoked				
Mean (SD)	39.85 (15.27)	35.86 (9.19)	53.90 (20.85)	51.00 (32.14)
Median (Range)	36.00 (21.50, 88.00)	35.50 (21.50, 58.00)	49.50 (34.00, 82.50)	35.00 (30.00, 88.00)
FEV1% Predicted				
Mean (SD)	86.32 (23.46)	85.88 (25.08)	90.00 (14.68)	84.00 (27.18)
Median (Range)	91.00 (29.00, 127.00)	91.00 (29.00, 127.00)	83.00 (74.00, 108.00)	91.00 (54.00, 107.00)
FEV1/FVC (%) Actual				
Mean (SD)	71.50 (13.97)	72.69 (13.37)	69.40 (11.28)	64.67 (25.15)
Median (Range)	75.50 (35.00, 92.00)	76.00 (35.00, 92.00)	71.00 (51.00, 82.00)	75.00 (36.00, 83.00)
**COPD (FEV1/FVC) < 0.7**	8 (24%)	6 (23%)	1 (20%)	1 (33%)
Max Histology, Baseline				
No Dysplasia	15 (44%)	11 (42%)	4 (80%)	0 (0%)
Mild Dysplasia	0 (0%)	0 (0%)	0 (0%)	0 (0%)
Moderate Dysplasia	14 (41%)	11 (42%)	1 (20%)	2 (67%)
Severe Dysplasia	5 (15%)	4 (15%)	0 (0%)	1 (33%)
Carcinoma *In Situ*	0 (0%)	0 (0%)	0 (0%)	0 (0%)

N, Number of participants in each group.

### Adverse events

Inhaled iloprost was well tolerated during both treatment schedules. Four participants, two from Cohort A and two from Cohort B, dropped out due to grade 2 toxicities (cough, headache and nausea, dyspnea, and wheezing). Two other individuals from Cohort A were removed for reasons unrelated to toxicity: pulmonary embolism requiring anticoagulation and hesitancy to take an additional medication. No unexpected toxicities were noted; there were no adverse events higher than grade 2 in either iloprost or placebo groups (**Supplementary Table 1**).

### Compliance

The I-neb AAD device records inhalation treatments to determine compliance. Treatment time ranged from 6 to 20 min with an average of 11 min. Total compliance, measured as mean percent of scheduled doses actually administered, across groups was Cohort A = 67.2% (Min–Max, 19%–98.5%); Cohort B = 70.2% (Min–Max, 13.4%–101%); placebo = 93.3% (Min–Max, 87.9%–99.2%). The predetermined definition of fully compliant was 80%; 46.2% of Cohort A, 60.0% of Cohort B, and 100% of placebo participants had ≥80% of scheduled doses actually administered and thus met the definition of fully compliant.

### Histology

Baseline bronchoscopy was performed in 34 individuals. A total of 19 had at least one biopsy graded as dysplastic (0 = mild; 14 = moderate; 5 = severe). Sputum cytology prior to bronchoscopy was completed on 33 individuals, including 2 of the 3 who qualified for the study based on a previous dysplastic biopsy. Of the 33 participants with mild or worse sputum atypia, 18 had one or more dysplastic biopsies. The fractions of dysplastic biopsies for varying grades of sputum atypia were as follows: normal, 1/1; mild, 9/18; moderate, 8/13; and severe, 1/2.

**Figure 2:**
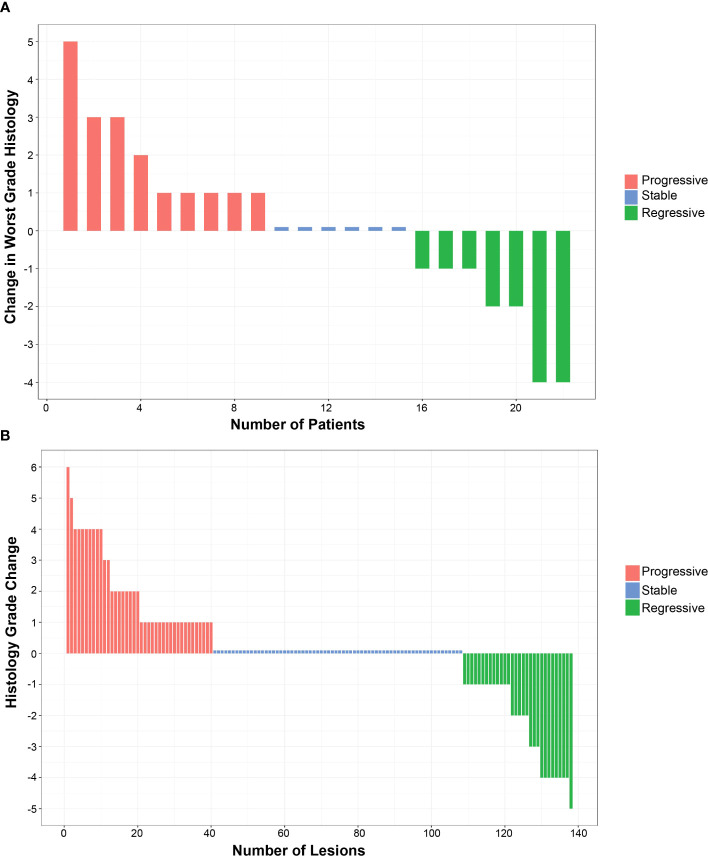
Effect of inhaled iloprost on histology. **(A)** Change in the worst histology post-iloprost per participant from Cohort **(A, B)** Change in all histology post-iloprost from a total 138 biopsies of participants at QID dose.

Although the trial was not powered to assess histologic response, the predetermined endpoint for treatment effect on histology was the change in maximum histologic score between baseline and final bronchoscopy (**Table 2**). Additional predetermined endpoints were change in mean histologic score and the dysplasia index, defined as fraction of biopsy sites scored as dysplastic. The early termination of enrollment into the placebo group and Cohort B limited size to three evaluable participants each, making comparison to Cohort A unlikely to be meaningful. Cohort A (22 evaluable participants) showed small and insignificant worsening of maximum (0.130, *p* = 0.79) and mean (0.08, *p* = 0.66) dysplasia scores. The dysplasia index showed a small, but not significant, improvement (−7.7%, *p* = 0.51). An alternative scoring system for grading response was assessed after study completion and results are shown in **Supplementary Table 2** (23). Among 22 individuals in Cohort A who had both bronchoscopies, there were *n* = 138 locations that were biopsied at both time points. There was no significant change in the worst grade histology in this group (**Figure 2A**). At the lesion level, 68 (49%) had no change in histologic grade (stable), whereas progression occurred in 40 (29%, progressive) and regression occurred in 30 (22% regressive) (**Figure 2B**), with no statistically significant change (*p* = 0.513, Stuart–Maxwell test of marginal homogeneity).

**Table 2:** Change in histology post iloprost and placebo.

Characteristic	Cohort A (Iloprost QID)				Cohort B (Iloprost BID)				Placebo			
	Visit 1 *N* = 26	Visit 2 *N* = 22	Change *N* = 22	*p*-value *^1^*	Visit 1 *N* = 5	Visit 2 *N* = 3	Change *N* = 3	*p*-value *^2^*	Visit 1 *N* = 3	Visit 2 *N* = 3	Change *N* = 3	*p*-value *^3^*
Maximum Histology (numeric)				0.79				0.50				0.37
Mean (SD)	3.8 (1.8)	3.9 (2.0)	0.1 (2.1)		2.4 (1.7)	4.7 (3.2)	2.7 (3.2)		5.3 (0.6)	3.0 (2.0)	-2.3 (2.5)	
Median (Range)	5.0 1.0, 6.0)	4.5 (1.0, 7.0)	0.0 (−4.0, 5.0)		2.0 (1.0, 5.0)	6.0 (1.0, 7.0)	4.0 (−1.0, 5.0)		5.0 (5.0, 6.0)	3.0 (1.0, 5.0)	−2.0 (−5.0, 0.0)	
Dropped out	0	4	4		0	2	2					
Mean Histology (numeric)				0.66				0.50				0.25
Mean (SD)	2.0 (1.0)	2.1 (0.9)	0.1 (0.8)		1.4 (0.7)	1.9 (1.0)	0.8 (0.9)		2.0 (0.3)	1.5 (0.5)	−0.5 (0.6)	
Median (Range)	1.8 (1.0, 4.9)	2.1 (1.0, 4.0)	0.0 (−1.8, 1.7)		1.2 (1.0, 2.7)	1.8 (1.0, 3.0)	0.8 (−0.2, 1.7)		2.1 (1.7, 2.2)	1.4 (1.0, 2.0)	−0.3 (−1.2, −0.1)	
Dropped out	0	4	4		0	2	2					
Dysplasia Index (DI)				0.51				0.37				0.17
Mean (SD)	0.2 (0.2)	0.2 (0.2)	0.0 (0.2)		0.1 (0.2)	0.2 (0.1)	0.2 (0.1)		0.2 (0.0)	0.1 (0.1)	−0.1 (0.1)	
Median (Range)	0.2 (0.0, 0.9)	0.2 (0.0, 0.8)	0.0 (−0.3, 0.4)		0.0 (0.0, 0.4)	0.2 (0.0, 0.3)	0.2 (0.0, 0.3)		0.2 (0.2, 0.2)	0.0 (0.0, 0.2)	−0.2 (−0.2, 0.0)	
Dropped out	0	4	4		0	2	2					

^1^Wilcoxon signed rank test with continuity correction.

^2^Wilcoxon signed rank exact test; Wilcoxon signed rank test with continuity correction.

^3^Wilcoxon signed rank test with continuity correction; Wilcoxon signed rank exact test.

N, Number of participants in each group.

One individual had a dramatic worsening of a biopsy site from normal to carcinoma *in situ*. A subsequent clinically indicated bronchoscopy 6 weeks later demonstrated regression to normal. This participant had an additional follow-up bronchoscopy 13 months later where the biopsy site showed reserve cell hyperplasia. A second participant had a site (carina between anterior and apico-posterior segments of the left upper lobe) that was not sampled on the baseline bronchoscopy that appeared visually abnormal on the final bronchoscopy and was read as carcinoma *in situ*. This site regressed to severe dysplasia at 3 months and to normal at 15-month follow-up bronchoscopies. In both situations, no treatment for the carcinoma *in situ* lesions occurred.

### Sputum cytology and inflammation score

The number of participants with pre- and post-treatment sputum cytology (**Supplementary Table 3**) was too small to discern any meaningful difference caused by iloprost treatment.

Endobronchial biopsies were scored for inflammation on a score from 0 (none) to 3 (severe). Changes in maximum and mean inflammation scores are shown for Cohort A, Cohort B, and placebo groups in **Supplementary Table 4**. For Cohort A, there was a small, statistically insignificant increase in both maximum (0.3) and mean (0.2) inflammation scores between baseline and final bronchoscopies.

### Effect on pulmonary function and symptoms

Results of PFTs, 6MWT, and SGRQ and CAT scores at baseline and 60-day visits are summarized in **Supplementary Tables 5**–**7**, and **8**, respectively. There were no statistically significant or clinically meaningful changes in pre-bronchodilator FEV1 or FVC, post-bronchodilator FEV1 or FVC, and 6MWT-associated measures including distance walked and dyspnea levels associated with the 6MWT, SGRQ, or CAT scores.

### Molecular analysis of iloprost effects on bronchial biopsies

**Figure 3:**
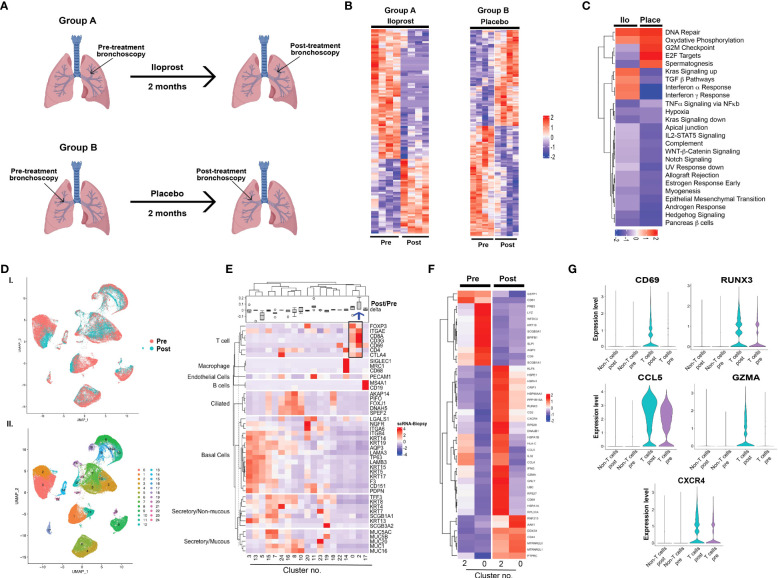
Molecular analysis of iloprost effects on bronchial biopsies. **(A)** Study design for bulk mRNAseq. **(B)** Heatmaps of differentially expressed genes pre- and post-iloprost and placebo samples. **(C)** Up- or downregulated pathways similar or different between iloprost and placebo. **(D)** UMAP presentation of integrated data from five pre- and post-biopsies used for 10x genomics single-cell sequencing and **(E)** unsupervised clustering showing 24 transcriptomic clusters. **(F)** Heatmap of various epithelial and non-epithelial cell types using known cell-type-specific markers. Delta represents ratio of post- and pre-cell numbers normalized by number of cells captured for each biopsy. Blue arrow shows cluster 2 that was expanded post-iloprost. **(G)** Heatmap of T cell clusters 0 and 2 pre- and post-treatment. **(H)** Violin plots showing genes that were significantly upregulated post-iloprost only in T cells.

Bulk mRNAseq and scRNAseq analysis were used to investigate iloprost-mediated changes in bronchial tissues. Considering the small size of the trial and to avoid molecular differences associated with varying histology grades, we restricted our analysis to histologically normal biopsies (grade 1.0) both at pre-treatment and 2 months post-iloprost (**Figure 3A**, group A). Four individuals from Cohort A were used in this analysis and were compared to two placebo participants (**Figure 3A**, group B). For each placebo participant, two biopsies with histology grade 1.0 at baseline and at 2 months bronchoscopy were included in the analysis. Heatmaps of genes significantly altered after iloprost or in placebo are shown in **Figure 3B**. A detailed list of these genes is provided in **Supplementary Tables 9A, B**. GSEA was used to identify pathways that are (i) upregulated both in placebo and in iloprost, (ii) downregulated both in iloprost and in placebo, (iii) upregulated only in iloprost, and (iv) downregulated only in iloprost (**Figure 3C**). This analysis plan was devised to recognize pathways involved in normal repair after bronchoscopy and biopsy (i and ii) or involved in iloprost exposure (iii and iv). DNA repair and oxidative phosphorylation were upregulated in both placebo and iloprost groups, while Wnt-β catenin, Notch signaling, IL2-Stat5, and EMT pathways were downregulated in both groups. Pathways that were upregulated by iloprost only included Kras signaling, TGFβ, and interferon α and γ response pathways, while pathways involved in cell proliferation such as G2M checkpoint, E2F targets, and spermatogenesis were downregulated in iloprost compared to placebo (normalized *p* < 0.01).

scRNAseq was performed using five paired biopsies (pre- and post-treatment from the same sites) from two participants, all with histology grade 1.0 both pre-treatment and post-iloprost. No placebo samples were available for scRNAseq. A total of 74,519 cells from the 10 biopsies were profiled. UMAP for dimensionality reduction of single-cell data and unsupervised clustering showed small differences in overall cellular distributions in pre- and post-treatment samples (**Figures 3D, E**; **Supplementary Figure 1A**). Known cell-type-specific markers for epithelial and non-epithelial (including immune) cells identified cell-type-specific clusters and their relative proportions in pre- and post-biopsies (**Figure 3F**). The main difference observed was an expansion of CD8-positive T cells (cluster 2) and a decrease in a subset of basal cells (cluster 5) post-iloprost. More in-depth analysis of cluster 5 identified only five genes (MALAT1, HBB, DDX17, SYEN2, and F3) that were exclusive to this cluster and were absent in the other basal cell clusters 3 and 13 (**Supplementary Figure 1B**). In contrast, T-cell clusters (clusters 0 and 2) in pre- and post-iloprost treatment identified a list of genes that are upregulated post-iloprost (**Figure 3G**). Significantly, key genes involved in interferon <υ>γ</υ> signaling including CD69, RUNX3, CCL5, Granzyme A (GZMA), and CXCR4 were upregulated by iloprost specifically in T cells compared to non-T cells (**Figure 3H**).Correlation between *in vitro* and *in vivo* responses to iloprost

**Figure 4:**
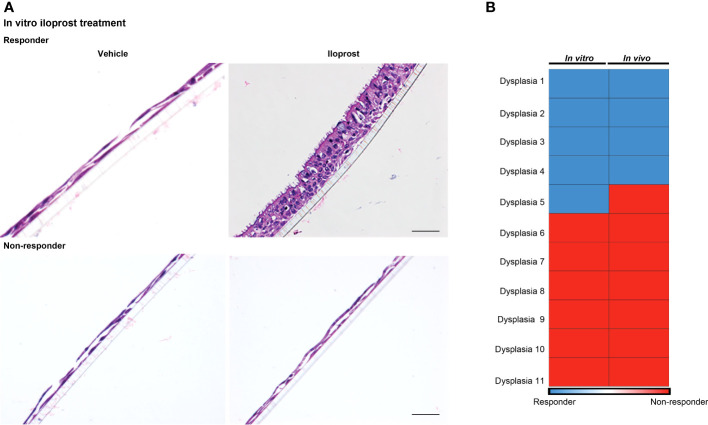
Correlation between in vitro and in vivo responses to iloprost. **(A)** H & E-stained membrane inserts of basal epithelial cells treated with either vehicle (left) or 1 μM iloprost (right). Responder culture (top) established a mucociliary epithelium and non-responder did not. A scale bar of 50 μm is applicable for all four panels. **(B)** Four of the five dysplasias that responded to iloprost *in vitro* also responded *in vivo*, and all six non-responders *in vitro* did not respond *in vivo*.

Eleven dysplastic biopsies (three severe and eight moderate dysplasia) from 10 individuals collected at pre-treatment bronchoscopy were successfully cultured at ALI with vehicle or iloprost and evaluated for response. Cells from 5 (2 severe and 3 moderate dysplasia) of the 11 biopsies responded to iloprost but not vehicle by re-establishment of a normal mucociliary epithelium, while 6 (1 severe and 5 moderate dysplasia) were unresponsive (**Figure 4A**; **Supplementary Figure 2**). Of the five biopsy sites that yielded cell cultures that responded to iloprost but not vehicle *in vitro*, four responded to inhaled iloprost by improving histology to a non-dysplastic grade. Of the six biopsy sites that yielded cell cultures that did not respond *in vitro*, none responded to inhaled iloprost by improving histology [agreement between response *in vivo* and *in vitro*, kappa coefficient 0.81 (95% CI 0.47, 1, **Figure 4B**)]. All of these participants were on the QID dosing and administered most (87.5% ± 9% for responders and 63.7% ± 29% for non-responders) of their scheduled iloprost doses.

## Discussion

Prostacyclin and its analog, iloprost, reduce tumor numbers dramatically in multiple preclinical chemical carcinogenesis models and improved bronchial dysplasia in approximately 50% of former smokers in a phase II trial of oral iloprost (4, 5, 8). The lack of availability of oral iloprost limited further human studies and thus we report on inhaled iloprost, an FDA-approved treatment for pulmonary hypertension.

Inhaled iloprost was well tolerated with minor, clinically expected toxicities related to its vasodilator activity. However, compliance, a secondary objective, was suboptimal with participants in the QID dosing group taking only 67.2% of scheduled doses. The i-Neb AAD device is the only FDA-approved means for administering inhaled iloprost, but it is significantly more time-consuming to use than standard metered dose inhalers used for asthma and COPD treatment. It is also likely that the minor side effects (headache and flushing) of inhaled iloprost may have reduced compliance.

Although the study was not statistically powered to detect treatment response of histologic endpoints to inhaled iloprost, exploratory analysis showed no significant change in any of the predetermined histologic endpoints. In a *post-hoc* analysis, we also assessed per-participant and per-lesion responses by a different set of response criteria used in previous trials (23) and did not find any significant response by these criteria either. Several unanticipated challenges were experienced during the trial that provide lessons for future interception trials. The loss of availability of placebo during the trial necessitated a change to an open label design. Consistent monitoring of the expiration of both study agent and placebo prior to trial initiation and during its conduct might have avoided this obstacle. The difficulties and expense of obtaining matched placebo for clinical trials is a well-known obstacle in the cancer prevention field (24).

Sputum cytologic atypia was utilized as a means to enrich for histologic dysplasia in study participants. Only 52% of participants with sputum atypia of mild or worse had histologic dysplasia detected on bronchoscopy, underscoring the need for improved methods to identify study populations with dysplasia. Obtaining and analyzing sputum prior to enrollment is a time-consuming process and improved methods to identify participants with endobronchial dysplasia for similar trials are needed. A highly multiplexed three-dimensional analytic system adapted to the evaluation of sputum for abnormal cells has been developed, and it or similar technologies may improve the performance of sputum cytology (10). Serum CRP levels have been reported to predict dysplasia progression and similar proteomic or metabolomic biomarkers may be useful for identifying participants with endobronchial dysplasia (25). However, attempts to operationalize CRP assessment prior to bronchoscopy did not improve trial efficiency in our previous experience (23).

The current trial design with repeated bronchoscopic biopsies at relatively short intervals gave rise to methodologic concerns. One individual progressed to carcinoma *in situ* between the initial and final biopsies and a second had carcinoma *in situ* at a site not previously biopsied on final bronchoscopy. Subsequent clinically indicated bronchoscopies demonstrated regression to non-dysplastic scores in both. We have observed an additional individual who developed carcinoma *in situ* 2 months after an initial bronchoscopy and then subsequently regressed to non-dysplastic histology at the same biopsy site in a different trial while we have never seen this in over 300 individuals who have participated in previous trials with a 6-month interval between bronchoscopies. Other explanations, including sampling error at an initial bronchoscopy with removal of the entire dysplastic lesion at the 2-month bronchoscopy and effects of a 2-month inhalation regimen, are possible. We caution that a 2-month interval between bronchoscopies may be too short to allow for resolution of inflammation and epithelial remodeling in some individuals.

Prostacyclin acts through a single-cell surface receptor, IP, and also binds and activates the nuclear receptor peroxisome proliferator-activated receptor (PPAR) gamma. Experiments with mice overexpressing PGIS and null for IP receptor demonstrated that the anti-tumor effect is independent of IP receptor (6). In a separate mouse model, PPAR gamma overexpression conferred a chemopreventive effect that was not augmented by exogenous iloprost, suggesting that prostacyclin and its analog iloprost act by activating PPAR gamma (6). However, a phase IIb trial of the PPAR gamma agonist pioglitazone failed to show efficacy in improving dysplasia, suggesting that iloprost’s mechanism is more complicated than solely PPAR gamma activation (26).

We further explored the molecular effects of inhaled iloprost on paired bronchial biopsies taken at the beginning and end of the trial using bulk mRNA sequencing. While limited by small numbers, this analysis identified signaling pathway similarities and differences between iloprost and placebo. Importantly, pathways upregulated after iloprost, but not placebo, included Kras signaling, interferon α, and interferon γ, all associated with immune response. Pathways involved in cell proliferation including G2M checkpoint, E2F targets, and spermatogenesis were similarly downregulated. Identification of Hallmark_spermatogenesis pathway in bronchial epithelial culture was confusing, but more in-depth analysis revealed that genes involved in this pathway included markers of proliferation such as CDK1, CCNA1, CCNB2, CDKN3, AurkA, AurkB, and Bub1, as well as markers of tubulin complex, mitotic spindle, and intracellular filaments such as GSTM3, PGK2, AKAP4, PACRG, ODF1, TEKT2, MLF1, SPATA6, and IFT88. We assume that the ciliary architecture of both bronchial epithelium and testis led to this nomenclature.

We also performed a limited number of scRNAseq analyses, demonstrating an increase in CD8 T cells (cluster 2) that also expressed CD69, a marker of tissue-resident memory cells (27). Genes upregulated in cluster 2 are involved in interferon γ signaling, TNF α, and allograft rejection, suggesting an immunomodulatory role for iloprost, as has been shown in mouse models of pulmonary hypertension and lung carcinoma (28–30). While intriguing, these scRNAseq and bulk RNAseq are highly preliminary findings based on a small number of individuals and biopsies. Owing to the small cohort size and multiple levels of dysplasia, we were unable to match basal histologic levels for gene expression analysis in responders and non-responders. Therefore, the only comparison we could reasonably make was between normal histology pre- and post-iloprost. The lack of a placebo group for scRNAseq limits interpretation as to which effects were due to a previous bronchoscopy with biopsy and which were related to iloprost. A well-controlled larger study is warranted for validation.

Perhaps the most significant finding of this study is the correlation between *in vitro* and *in vivo* response. Of the 11 dysplasias that were tested for iloprost response *in vitro*, all but one accurately predicted clinical response. One individual had two different dysplastic sites biopsied and cultured; both responded *in vitro* but only one responded *in vivo* while the other did not. This underscores the fact that each dysplasia may be biologically unique and may support a precision approach for cancer prevention. As *in vitro* culture only contains epithelial cells, this finding also demonstrates that iloprost directly affects epithelial cells to reverse dysplasia. The scRNAseq finding that iloprost altered a CD8 T-cell population *in vivo* supports additional potential mechanisms of action. Further studies are needed to confirm and extend these findings.

In conclusion, inhaled iloprost was tolerable at the dose levels tested, but compliance was suboptimal. Thus, there are insufficient data at the current time to support further inhaled iloprost trials. Significant challenges were encountered in terms of participant compliance, identification of study participants with dysplasia, interpretation of biopsy results in the setting of a short interval between bronchoscopies, and the unanticipated expiration of placebo. Molecular studies suggest new insights into the effects of iloprost on the airway mucosa. The response to iloprost by epithelial progenitors cultured at the ALI may be a promising avenue to pursue in order to develop predictive biomarkers should oral iloprost become available again, although this approach will need to be operationalized prior to larger clinical trials. Whether the progenitor ALI culture model will be applicable to other chemopreventive interventions deserves further study.

## Data availability statement

This manuscript contains transcriptomic data that included both bulk and single cell RNA sequencing. All the raw data has been submitted to GEO (Gene expression omnibus) database that is maintained by NCBI. The accession number assigned to this data is GSE240002. https://www.ncbi.nlm.nih.gov/geo/query/acc.cgi?acc=GSE240002 is the URL associated with this data. The raw sequencing data will be publicly released on July 20th, 2024.

## Ethics statement

The studies involving human participants were reviewed and approved by University of Colorado Multiple Institutional Review Board. The patients/participants provided their written informed consent to participate in this study.

## Author contributions

YM: Conceptualization, trial design, enrollment, bronchoscopy, data analysis, manuscript writing; MG: Conceptualization, experiments, data analysis, manuscript writing; DM: Pathology, data analysis; BK: Enrollment, protocol administration, data analysis; ES: Conceptualization, trial design, data analysis, manuscript writing; LB: trial design, data analysis; MK: Study statistician; IH: Study statistician; KB: Trial administrator; TS: Trial administrator; JK: Bioinformaticians; HK Bioinformaticians; DH: data analysis; SK: Conceptualization, trial design, data analysis; RK: Conceptualization, trial design, enrollment, bronchoscopy, data analysis. All authors contributed to the article and approved the submitted version.
