# Supplementary material for: Phase Ib trial of inhaled iloprost for the prevention of lung cancer with predictive and response biomarker assessment
Source: Front Oncol. 2023 Aug 30;13:1204726. doi: 10.3389/fonc.2023.1204726 (PMC10499515; doi:10.3389/fonc.2023.1204726)
Supplement: Supplementary file 1 [file DataSheet_1.pdf]

Title: Phase Ib trial of inhaled iloprost for the prevention of lung cancer with predictive and response biomarker assessment.

York E Miller, Moumita Ghosh*, Daniel T Merrick, Brandi Kubala, Eva Szabo, Lisa Bengtson, Masha Kocherginsky, Irene B. Helenowski, Kelly Benante, Tia Schering, Jiyhe Kim, Hyunmin Kim, Duc Ha, Raymond C Bergan, Seema A Khan and Robert L. Keith

Supplemental Tables and Figures:

Supplemental Figure 1

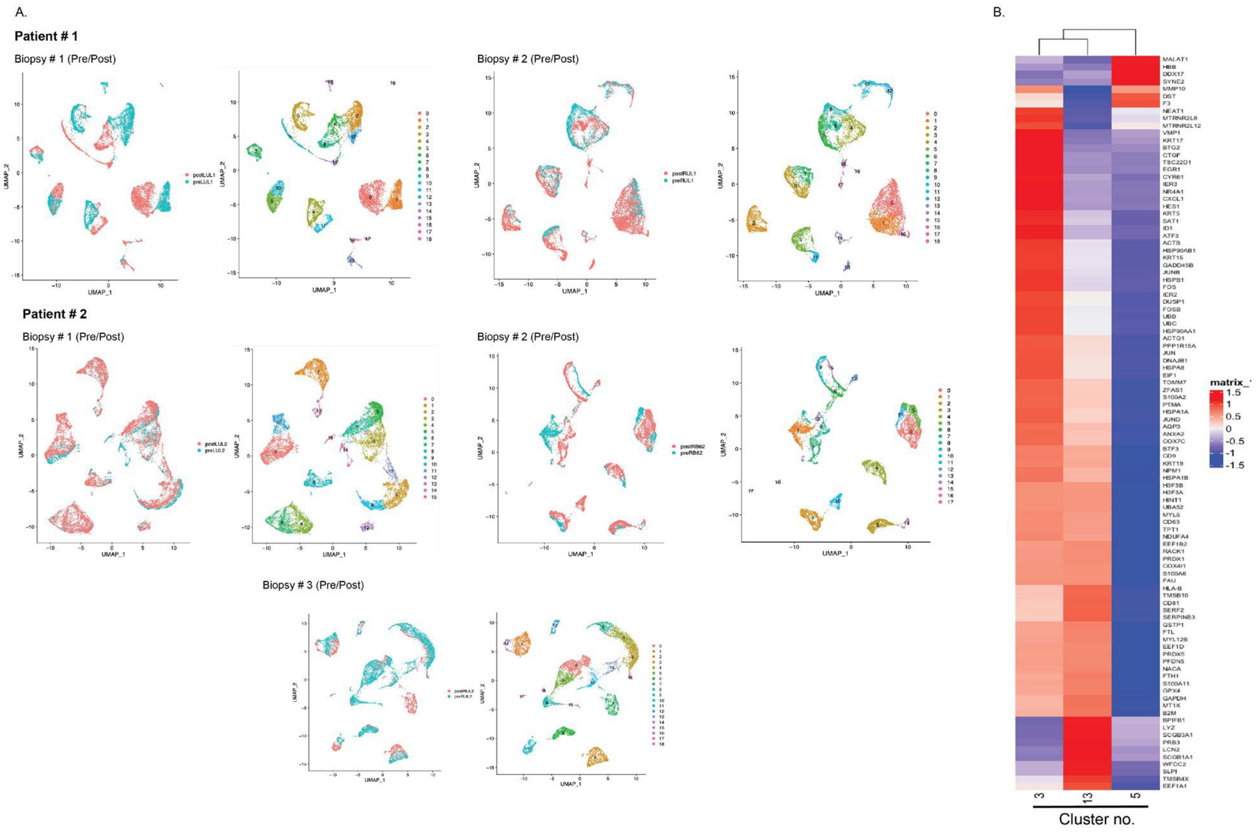

Single cell RNAseq (sc) of biopsies pre and post iloprpst. (A) UMAP analysis of visualization of scRNAseq data for pre-post analysis of five biopsies from two study participants. (B) Heatmap to identify basal cell genes impacted by iloprost comparing clusters 3, 13 and 5.

Supplemental Figure 2

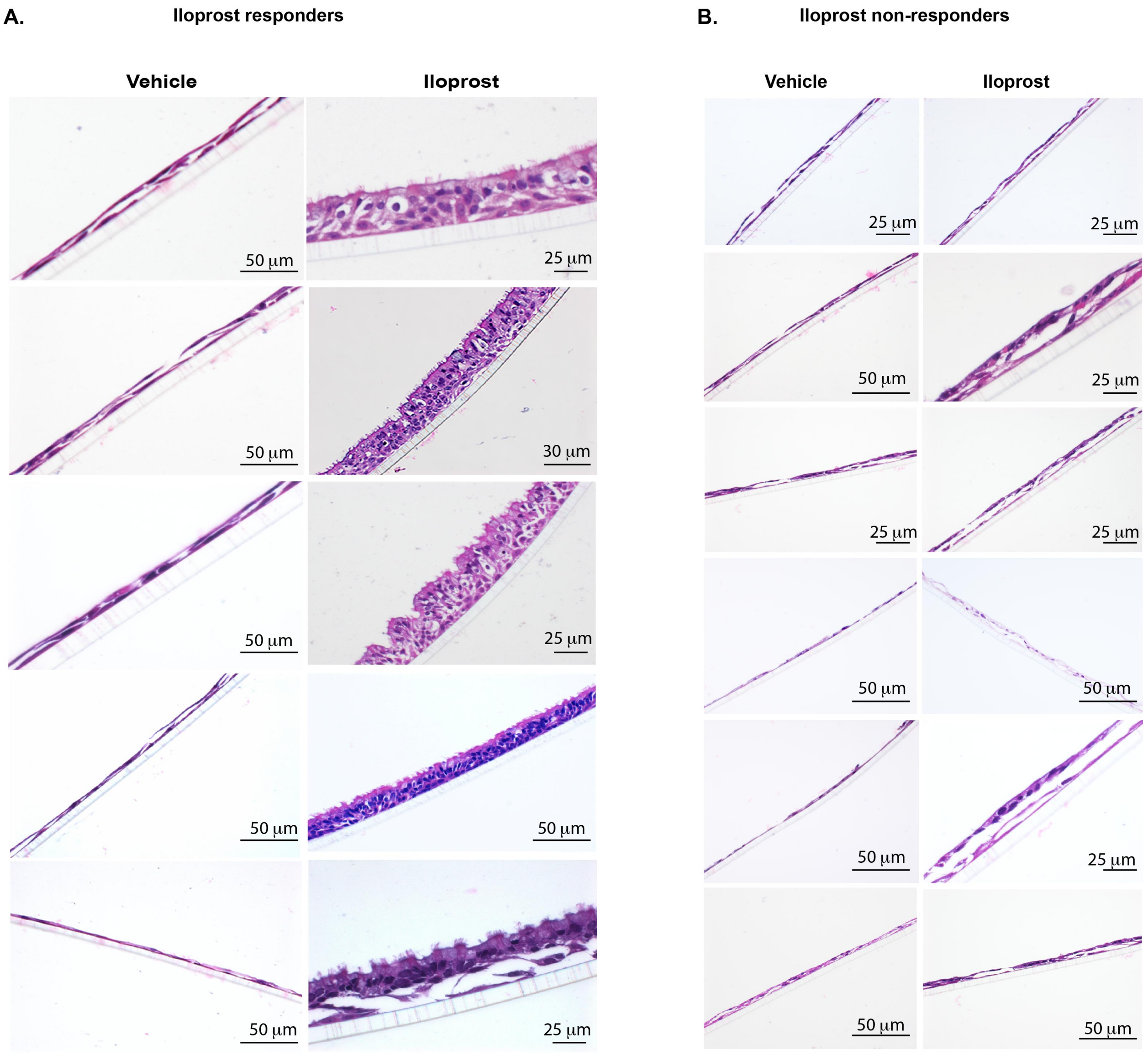

Airway epithelial culture system to determine iloprost response *in vitro*. H & E stained images of (A) five basal progenitor cultures that responded to iloprost in vitro and (B) six non-responders that did not respond. Scale bars are added in each panel.

Supplemental Table 1. Adverse events summary.

**Adverse Event** (AE)	**Grade 1 (Cohorts A+B)** (n=31)	**Grade 1 Placebo** (n=3)	**Grade 2 (Cohorts A+B)** (n=31)	**Grade 2 Placebo** (n=3)
Headache	12	1	3	0
Cough	12	2	3	0
Productive cough	2	0	1	0
Nausea	1	0	1	0
Dyspnea	4	0	1	0
Wheezing	0	0	1	0
Flushing	25	2	0	0
Dizziness	11	2	0	0

Adverse events possibly, probably or definitely related to study treatment (Cohorts A+B combined).

Supplemental Table 2. Histological response per participant and per lesion.

A. Per Participant Response.

Variable	**Cohort A N = 22**	**Cohort B N = 3**	**Placebo N = 3**
CR	2 (7.69%)	0 (0.00%)	1 (33.33%)
PR	1 (3.85%)	0 (0.00%)	0 (0.00%)
SD	0 (0.00%)	0 (0.00%)	2 (66.67%)
PD	12 (46.15%)	2 (40.00%)	0 (0.00%)
no dysplasia at baseline or f/u	6 (23.08%)	1 (20.00%)	0 (0.00%)
Missing	5 (19.23%)	2 (40.00%)	0 (0.00%)

N = Number of patients per group
CR: Complete Response
PR: Partial Response
SD: Stable Disease
PD: Progressive Disease

B. Per Lesion Response.

Variable	**Cohort A N = 172**	**Cohort B N = 33**	**Placebo N = 20**
CR	14 (8.1%)	0 (0%)	1 (5.0%)
SD	12 (7.0%)	0 (0%)	3 (15%)
PD	19 (11%)	3 (9.1%)	0 (0%)
metaplasia or lower in the per lesion response	94 (55%)	16 (48%)	15 (75%)
Missing	33 (19%)	14 (42%)	1 (5.0%)

N = Number of lesions per group
CR : Complete response
SD: Stable Disease
PD: Progressive Disease

Supplemental Table 3. Determination of sputum cytology.

A. VisionGate Fixative:

	Cohort A			Cohort B			Placebo		
Variable	Visit 1 N = 13	Visit 2 N = 13[1]	p-value[1]	Visit 1 N = 2	Visit 2 N = 2	p-value[2]	Visit 1 N = 2	Visit 2 N = 2	p-value[*]
(Worst score)	4.00 (4.00, 5.00)	4.50 (4.00, 5.00)	0.42	4.00 (4.00, 4.00)	5.00 (5.00, 5.00)	>0.99	5.50 (5.25, 5.75)	5.00 (5.00, 5.00)	
N/A	6	5		1	1		0	1	

[1]Median (IQR); n (%)
[2]Wilcoxon signed rank test with continuity correction
[3]Wilcoxon signed rank exact test

B. Saccomano Fixative:

	Cohort A			Cohort B			Placebo		
Variable	Visit 1 N = 15	Visit 2 N = 15[1]	p-value	Visit 1 N = 2	Visit 2 N = 2	p-value[2]	Visit 1 N = 2	Visit 2 N = 2	p-value*
(Worst score)	5.00 (5.00, 5.00)	5.00 (4.25, 5.00)	>0.99	5.00 (5.00, 5.00)	3.00 (3.00, 3.00)	>0.99	NA (NA, NA)	4.00 (4.00, 4.00)	
N/A	6	9		1	1		2	1	

[1]Median (IQR); n (%)
[2]Wilcoxon signed rank exact test

N = Number of participants in each group
N/A = Not available
p[1 and 2] = values from the signed rank test of changes from visit 1 to visit 2, by cohort
p* = Only 2 participants

Supplemental Table 4. Inflammation identified on biopsies pre and post iloprost.

Characteristic	Overall, N = 34	Cohort A Iloprost QID, N = 22	Cohort B Iloprost BID, N = 3	Placebo, N = 3
**Max Inflammation, Visit 1**				
Absent	9 (26%)	8 (31%)	1 (20%)	0 (0%)
Mild	19 (56%)	15 (58%)	2 (40%)	2 (67%)
Moderate	6 (18%)	3 (12%)	2 (40%)	1 (33%)
**Max Inflammation, Visit 2**				
Absent	4 (14%)	4 (18%)	0 (0%)	0 (0%)
Mild	14 (50%)	10 (45%)	1 (33%)	3 (100%)
Moderate	10 (36%)	8 (36%)	2 (67%)	0 (0%)
Dropped out	6	4	2	0
**Worst grade Inflammation change**				
Mean (SD)	0.25 (0.75)	0.27 (0.70)	0.67 (1.15)	-0.33 (0.58)
Median (Range)	0.00 (-1.00, 2.00)	0.00 (-1.00, 2.00)	0.00 (0.00, 2.00)	0.00 (-1.00, 0.00)
Dropped out	6	4	2	0
**Mean Inflammation, Visit 1**				
Mean (SD)	0.36 (0.33)	0.32 (0.31)	0.36 (0.30)	0.65 (0.50)
Median (Range)	0.33 (0.00, 1.17)	0.31 (0.00, 1.17)	0.33 (0.00, 0.67)	0.62 (0.17, 1.17)
**Mean Inflammation, Visit 2**				
Mean (SD)	0.53 (0.42)	0.55 (0.44)	0.74 (0.25)	0.20 (0.03)
Median (Range)	0.43 (0.00, 1.50)	0.43 (0.00, 1.50)	0.71 (0.50, 1.00)	0.20 (0.17, 0.22)
Dropped out	6	4	2	0
**Mean Inflammation change**				
Mean (SD)	0.14 (0.43)	0.18 (0.38)	0.40 (0.28)	-0.46 (0.52)
Median (Range)	0.17 (-1.00, 0.89)	0.21 (-0.60, 0.89)	0.33 (0.17, 0.71)	-0.40 (-1.00, 0.03)
Dropped out	6	4	2	0

Supplemental Table 5. Lung function parameters pre and post iloprost.

	Cohort A			Cohort B			Placebo		
Variable	Visit 1<br>N = 26[1]	Visit 2<br>N = 22[1]	p-value[2]	Visit 1<br>N = 5[1]	Visit 2<br>N = 3[1]	p-value[3]	Visit 1<br>N = 3[1]	Visit 2<br>N = 3[1]	p-value[3]
Spirometry: FVC (L) Pre-bronchodilator: Actual	3.76 (2.86, 4.75)	3.60 (2.51, 4.67)	0.17	4.20 (3.87, 4.49)	4.48 (4.13, 4.92)	>0.99	4.40 (4.20, 4.65)	4.34 (4.17, 4.39)	>0.99
N/A	0	4		0	2				
Spirometry: FVC (L) Post-bronchodilator: Actual	3.84 (2.96, 4.56)	3.52 (3.02, 4.09)	0.91	4.28 (3.58, 4.77)	4.55 (4.26, 4.84)	0.50	4.37 (4.15, 4.77)	4.50 (4.20, 4.80)	>0.99
N/A	3	7		1	3				
Spirometry: FEV1 (L) Pre-bronchodilator: Actual	2.84 (1.81, 3.77)	2.44 (1.73, 3.51)	0.056	2.77 (2.14, 3.45)	3.57 (3.02, 3.59)	0.75	3.28 (2.51, 3.30)	3.17 (2.32, 3.23)	0.25
N/A	0	4		0	2				
Spirometry: FEV1 (L) Post-bronchodilator: Actual	3.02 (1.81, 3.64)	2.49 (1.91, 3.45)	0.84	3 (2, 3)	141 (72, 210)	>0.99	3.32 (2.67, 3.38)	3.25 (2.54, 3.30)	0.25
N/A	3	7		1	3				
Diffusion: DLCOunc (ml/min/mmHg) Pre-bronchodilator: Actual	30 (21, 34)	29 (22, 34)	0.059	19 (17, 33)	24 (19, 27)	0.25	43 (30, 44)	36 (25, 41)	0.25
N/A	0	4		0	2				

Variable	Cohort A Visit 1 N = 26[1]	Cohort A Visit 2 N = 22[1]	Cohort A p-value[2]	Cohort B Visit 1 N = 5[1]	Cohort B Visit 2 N = 3[1]	Cohort B p-value[3]	Placebo Visit 1 N = 3[1]	Placebo Visit 2 N = 3[1]	Placebo p-value[3]
Spirometry: FVC (L) Post vs. pre-bronchodilator: Actual	0.12 (-0.07, 0.31)	0.16 (-0.01, 0.41)	0.50	-0.03 (-0.06, 0.14)	-0.02 (-0.13, 0.09)	>0.99	-0.03 (-0.05, 0.11)	0.05 (-0.03, 0.40)	0.50
N/A	3	7		1	3				
Spirometry: FEV1 (L) Post vs. pre-bronchodilator: Actual	0.18 (0.04, 0.22)	0.22 (0.12, 0.32)	0.12	0 (0, 0)	138 (69, 207)	>0.99	0.16 (0.07, 0.22)	0.17 (0.06, 0.27)	0.75
N/A	3	7		1	3				
Gold score	5.00 (5.00, 5.00)	5.00 (5.00, 5.00)	>0.99	5.00 (4.00, 5.00)	5.00 (4.50, 5.00)	>0.99	5.00 (4.50, 5.00)	5.00 (4.50, 5.00)	
N/A	0	4		0	2				

[1]Median (IQR)
[2]Wilcoxon signed rank test with continuity correction; Wilcoxon signed rank exact test
[3]Wilcoxon signed rank exact test; Wilcoxon signed rank test with continuity correction

Supplemental Table 6. Level of dyspnea pre and post iloprost.

	Cohort A			Cohort B			Placebo		
Variable	Visit 1 N = 26[1]	Visit 2 N = 22[1]	p-value[2]	Visit 1 N = 5[1]	Visit 2 N = 3[1]	p-value[3]	Visit 1 N = 3[1]	Visit 2 N = 3[1]	p-value[3]
Distance (m)	321.87 (273.59, 370.15)	321.87 (305.77, 354.05)	0.83	304.17 (232.55, 313.02)	241.40 (209.21, 273.59)	0.75	358.14 (340.00, 364.14)	358.88 (308.19, 372.56)	>0.99
N/A	6	1		2	0				
rest dyspnea	0.00 (0.00, 0.00)	0.00 (0.00, 0.00)	>0.99	0.00 (0.00, 0.00)	0.00 (0.00, 0.00)		0.00 (0.00, 0.00)	0.00 (0.00, 0.00)	
N/A	6	1		2	0				
6 minutes dyspnea	0.00 (0.00, 0.50)	0.00 (0.00, 0.50)	0.59	0.00 (0.00, 0.03)	0.00 (0.00, 0.00)	>0.99	0.00 (0.00, 0.25)	0.00 (0.00, 0.25)	
N/A	6	1		2	0				
6 minutes - rest dyspnea	0.00 (0.00, 0.50)	0.00 (0.00, 0.50)	0.61	0.00 (0.00, 0.03)	0.00 (0.00, 0.00)	>0.99	0.00 (0.00, 0.25)	0.00 (0.00, 0.25)	
N/A	6	1		2	0				

[1]Median (IQR)
[2]Wilcoxon signed rank test with continuity correction
[3]Median (IQR)

Supplemental Table 7. Respiratory symptoms pre and post iloprost.

	Cohort A			Cohort B			Placebo		
Variable	Visit 1 N = 26[1]	Visit 2 N = 22[1]	p-value[2]	Visit 1 N = 5[1]	Visit 2 N = 3[1]	p-value[3]	Visit 1 N = 3[1]	Visit 2 N = 3[1]	p-value[3]
Symptom score	9.08 (0.00, 11.01)	0.00 (0.00, 9.34)	0.11	0.00 (0.00, 9.36)	0.00 (0.00, 0.00)	>0.99	0.00 (0.00, 4.64)	0.00 (0.00, 9.32)	>0.99
N/A	0	4		0	2				
Activity score	33.23 (18.90, 59.53)	32.64 (13.51, 62.93)	0.48	53.54 (41.77, 54.34)	53.54 (50.61, 53.94)	0.37	23.33 (14.77, 38.88)	29.63 (26.48, 41.62)	0.50
N/A	0	4		0	2				
Impact score	11.52 (5.54, 29.45)	9.63 (3.97, 27.79)	0.32	12.31 (10.85, 20.50)	8.32 (6.14, 8.99)	0.75	7.56 (3.78, 7.66)	0.00 (0.00, 6.23)	>0.99
N/A	0	4		0	2				
Total score	16.82 (9.17, 31.53)	17.33 (6.10, 34.82)	0.97	23.54 (23.01, 23.54)	18.87 (18.60, 20.23)	>0.99	12.62 (7.25, 16.62)	16.25 (11.66, 17.47)	0.50
N/A	0	4		0	2				

[1]Median (IQR)
[2]Wilcoxon signed rank test with continuity correction
[3]Wilcoxon signed rank test with continuity correction; Wilcoxon signed rank exact test

Supplemental Table 8

	Cohort A			Cohort B			Placebo		
Variable	Visit 1 N = 26[1]	Visit 2 N = 22[1]	p-value[2]	Visit 1 N = 5[1]	Visit 2 N = 3[1]	p-value[2]	Visit 1 N = 3[1]	Visit 2 N = 3[1]	p-value[2]
Total COPD score	13 (8, 21)	11 (9, 20)	0.49	13.00 (8.00, 14.00)	9.00 (7.00, 10.50)	0.59	11.00 (10.00, 12.00)	13.00 (9.50, 14.00)	>0.99
N/A	0	4		0	2				

[1]Median (IQR)
[2]Wilcoxon signed rank test with continuity correction

Supplemental Table 9A. Differentially expressed genes between pre and post iloprost.

Gene Name	logFC	AveExpr	t- Statistics	P.Value
RNY1	-5.648552	0.52169091	-13.373844	2.37E-05
PHACTR3	-1.3982806	-1.3869145	-10.912574	6.88E-05
POU4F3	-1.0667385	-1.7692159	-9.0381573	0.00018223
PRICKLE2-AS1	-0.3739512	-2.1156095	-8.5029017	0.00024883
VAMP3	0.17990318	3.83974871	7.47591581	0.00047618
FANCL	0.26637215	2.24891779	6.9720994	0.00067351
GFAP	-1.0075954	-1.7267874	-6.9661589	0.00067635
LOC653653	-1.0733442	-1.765913	-6.9455867	0.0006863
LOC101930452	0.44928791	-1.3688062	6.86843616	0.00072518
GIGYF2	-0.0864452	2.60336406	-6.7312157	0.00080086
LOC102723766	-1.5154164	-1.2211914	-6.4937759	0.00095472
DDX12P	0.3248061	0.44751895	6.3975594	0.00102671
UMPS	0.11130984	1.63688897	6.35281709	0.00106233
LINC01031	-1.3755848	-1.6147927	-6.2830147	0.00112081
ZNF319	-0.1477296	1.87054637	-6.2295528	0.00116814
ZNF341-AS1	-1.6912666	-1.4569518	-6.1624233	0.0012309
TAS2R5	0.75698262	0.06856206	6.13766918	0.00125503
FLJ41200	0.89523934	-0.4398878	6.12640828	0.00126619
PER1	-0.8094836	3.10604104	-6.1214801	0.00127111
LINC00346	-0.5129082	-0.755316	-6.0085341	0.00139018
LOC101926933	-1.3005619	-1.6523041	-5.9387241	0.00147025
SEMA6C	0.3492003	1.49917563	5.80706838	0.00163632
CES3	-0.3800145	1.11431184	-5.7360424	0.00173491
LOC101928270	-2.1762291	-1.2144706	-5.6543109	0.00185699
HES5	-0.5979527	-1.9145511	-5.5707653	0.00199221

LOC93622	0.20188598	2.76911644	5.56320377	0.002005
VCX	-0.466698	-2.0692361	-5.3966397	0.00231241
LINC01344	-1.9385291	-0.8277943	-5.3838425	0.00233821
TRMT2B	-0.130562	1.92668178	-5.3673516	0.00237194
VPS37D	0.34737913	1.05406486	5.33714731	0.00243519
DLK1	-0.9488842	-1.828143	-5.2251521	0.00268737
WEE2	-0.8214572	-1.6671753	-5.1669324	0.00283032
C1orf53	-0.4261088	1.57475973	-5.1646423	0.00283612
POU5F1P5	-0.7653652	-1.9199025	-5.1586203	0.00285143
ARRDC1-AS1	0.2214684	2.29265478	5.13720914	0.00290667
RGS20	-1.0898835	-1.7576434	-5.1266054	0.00293448
FCN2	-1.3497686	-1.6277008	-5.112923	0.00297082
C12orf29	0.14962974	1.64265824	5.07694985	0.00306888
IGSF21	-1.0160447	-1.4050404	-5.0124981	0.00325404
LRRC4B	0.96750573	-0.0016429	5.00607142	0.0032732
HS3ST5	-0.5622574	-2.0214564	-5.0034424	0.00328108
GCNT3	-1.4229001	0.64558292	-4.9892816	0.00332387
ENPP6	0.40637818	-1.1608363	4.97575431	0.00336536
LARS2-AS1	-0.3122347	-2.1464677	-4.9390352	0.003481
LOC101928674	-1.5309786	-1.2928999	-4.9191699	0.00354547
PDC	-0.8452322	-1.879969	-4.9066521	0.0035868
NKX1-2	-1.7874862	-0.961199	-4.8917276	0.00363681
LOC101927164	-0.9241873	-1.5679133	-4.8801245	0.00367624
SREK1IP1	0.1746067	1.61861231	4.87592718	0.00369062
EXD3	0.31942274	2.13970781	4.84623948	0.00379424
HIST1H4H	-2.1790244	-1.2130729	-4.8316626	0.00384633
ZNF793-AS1	-0.5324835	0.67359248	-4.778485	0.00404349
ZNF22	0.25301923	2.68193958	4.77547799	0.00405498

SOWAHB	-0.4022867	1.02244996	-4.7634294	0.0041014
CASR	-0.6870859	-1.8730967	-4.7488313	0.00415846
HRH1	0.4597254	0.85350345	4.73859925	0.00419901
ZNF286A	0.20255026	0.8055988	4.7079925	0.00432301
TBC1D17	0.13956064	3.26305499	4.68145082	0.00443396
LMAN2L	0.16755757	2.23383026	4.67894744	0.0044446
VARS	-0.1078495	2.56404335	-4.6734818	0.00446791
IRF3	0.14794851	3.77147351	4.62475149	0.00468211
SLC9A6	-0.170786	1.57004177	-4.5952098	0.0048177
LOC339874	-0.8655711	-1.7621648	-4.5914645	0.00483521
PEF1	0.14358103	3.55242863	4.53905505	0.00508804
LINC01144	0.66234992	0.43688128	4.52821547	0.0051422
FAM222A	-0.5241207	0.8054314	-4.493877	0.0053182
LOC729080	-1.1670647	-1.7190527	-4.493076	0.00532238
LINC00669	1.21876466	-1.4306337	4.47745679	0.00540479
COLEC10	-1.0616953	-1.7717375	-4.4639435	0.00547726
CAGE1	-0.2799995	-2.120894	-4.4578508	0.0055103
CIRBP	0.152468	5.71725585	4.44532245	0.00557896
FAM66A	-1.2758944	-1.6646379	-4.4274636	0.00567852
FAM204A	0.25562776	2.16911217	4.41667123	0.00573968
LOC102723373	-1.8646402	-1.0276418	-4.4134546	0.00575805
CLEC9A	-0.9297602	-1.7701272	-4.4024789	0.00582125
SAE1	0.1097907	3.26056091	4.38408511	0.00592896
SHH	-0.8201748	0.57683753	-4.3757345	0.0059786
LINC00176	0.25361956	-0.7005975	4.35991652	0.00607395
C9orf142	0.2231371	3.47856117	4.34804692	0.00614664
ATP2B2	-0.0868363	-2.2591669	-4.3412375	0.00618879
SAA1	-0.6722954	5.14197941	-4.3377215	0.00621069

ASIC3	0.2894461	0.70522397	4.3281247	0.0062709
CECR5	0.07412679	2.67141039	4.32154828	0.00631254
LINC00996	-0.8499911	-1.466762	-4.2975438	0.00646725
LPO	-2.6362708	-0.0358279	-4.2974813	0.00646766
SPATA1	-0.9138297	-1.5355287	-4.2690665	0.00665644
C2orf68	0.20247707	2.30206403	4.22142574	0.00698723
PAAF1	-0.2062688	2.40411124	-4.1972583	0.00716218
GALNT5	-0.4613674	2.02461889	-4.1713624	0.00735521
ZBTB22	0.18997339	2.69626273	4.17133157	0.00735544
OR7C1	-0.9894683	-1.6669631	-4.1682807	0.00737857
GEMIN6	-0.2576245	2.6522886	-4.1673473	0.00738566
VNN1	-0.8026973	0.02143455	-4.1660208	0.00739576
AARS	-0.1298267	3.19840412	-4.1593922	0.00744643
ERMAP	0.11565939	2.40668194	4.15020037	0.00751736
CTU2	-0.0961017	2.10055623	-4.1252505	0.00771379
NME6	-0.2300645	1.9158415	-4.1252051	0.00771415
LINC01539	-0.5899483	-1.9451626	-4.1095281	0.00784057
SIPA1L1	-0.176009	2.69975387	-4.1061188	0.00786837
RUSC1	0.13075064	2.90419751	4.09805741	0.00793456
CNNM3	-0.1181859	2.48903955	-4.0131889	0.00867122
PQLC2	0.07829748	2.00688786	4.00835729	0.00871544
BAMBI	-0.258631	2.20806193	-4.0077165	0.00872133
EPHA6	-0.9852401	-1.6834919	-4.0020927	0.00877316
LOC100507334	-0.9408388	-1.7115147	-4.001955	0.00877443
MTBP	-0.356504	0.28778269	-3.982269	0.00895869
IL24	-0.7362309	-1.8354635	-3.9708145	0.00906792
FGFBP1	-1.509905	1.97707026	-3.9558533	0.00921288
PRND	1.16863403	-1.2315017	3.95244807	0.00924624

SKA2	0.19264456	2.34247479	3.94351473	0.0093344
TMEM5-AS1	-1.546264	-1.5294531	-3.9363618	0.00940569
CNGA1	0.65129939	-0.4193366	3.92530373	0.00951711
GUSB	0.18237938	3.35935646	3.90563871	0.00971897
GALT	0.29073342	2.55141732	3.9006123	0.00977134
FAM43B	-0.5622	-2.0214851	-3.892862	0.00985272
HCG11	0.25106276	1.64873809	3.88639845	0.00992118
ROPN1L-AS1	-0.8957414	-1.8547144	-3.8862634	0.00992262
C12orf71	-0.7472006	-1.9289848	-3.8799909	0.00998958
R3HCC1L	0.22198305	2.26132455	3.8718727	0.010077
HS1BP3-IT1	-0.9604323	-1.8223689	-3.8685439	0.0101131
EDN1	0.52239789	0.96698413	3.85530033	0.01025818
FANCD2	0.09433787	0.6417485	3.84184507	0.01040799
SPRR3	-1.5194214	-0.8673293	-3.8406105	0.01042186
PHF5A	0.11918578	3.25735478	3.82445047	0.01060535
LINC00881	-0.8693391	-1.6325673	-3.8170358	0.01069076
WIF1	-1.3740704	-0.9670304	-3.8061408	0.01081768
PLEKHA6	-0.2715909	2.071147	-3.7805197	0.01112291
HIST1H1B	-0.6101097	-1.9975303	-3.7658064	0.0113026
CFI	0.412207	3.13078755	3.76454325	0.01131817
ENTPD2	0.37671205	0.91010712	3.75541724	0.01143145
ZFAT	-0.1750732	1.02013644	-3.7544453	0.01144359
RAP2C	0.1956126	1.76954129	3.75019574	0.01149683
KRT17	-0.4550802	6.2438479	-3.7470545	0.01153637
TARS	0.05736731	2.83843521	3.74572873	0.01155311
ROPN1	-0.7080252	-1.9485725	-3.7445132	0.01156847
SPDEF	-0.4061373	3.20665321	-3.7414104	0.0116078
MTM1	-0.139048	2.04454265	-3.7353196	0.01168545

MPO	-0.7672574	-1.9189564	-3.7344645	0.01169639
SLC2A5	-0.9050272	-1.4334635	-3.7338544	0.01170421
FLJ36777	-0.7817357	-1.3805754	-3.7273977	0.01178731
MIR5047	-5.0089722	2.1353241	-3.7236339	0.01183606
PDK1	-0.4382482	0.52617853	-3.7209199	0.01187136
C2orf27B	0.29979974	-2.1526852	3.70929516	0.01202388
HSPA4	-0.2372142	3.31525961	-3.6983302	0.01216977
LUC7L3	0.35651827	4.52272476	3.68923665	0.01229228
ZSCAN22	0.30313772	0.28496954	3.67867495	0.01243632
IZUMO1	-0.6119206	-1.8466366	-3.6740787	0.01249959
TSPYL1	-0.1140083	3.55539024	-3.6616557	0.01267244
SMAD1	0.20851131	2.67170223	3.65928319	0.01270576
LINC00336	1.25213723	-1.4115707	3.64407635	0.01292167
SSTR5-AS1	-0.8195417	-1.749568	-3.6384711	0.01300229
JMJD7-PLA2G4B	-2.6027153	0.02831337	-3.6320754	0.01309497
HSD3B7	0.54861897	1.9200389	3.62407709	0.01321193
SGPP2	-0.3271287	2.98190208	-3.6020379	0.01354034
SGK2	0.50005477	0.09903778	3.59742149	0.01361029
CNTNAP1	0.45448406	0.83017439	3.59349511	0.01367011
GAPDHS	-1.1547267	-1.4299955	-3.5875801	0.01376077
FUS	0.10263631	3.85941387	3.58720085	0.01376661
NT5C1B	-0.5806534	-1.8766384	-3.5829217	0.01383266
GLMP	0.13607679	3.11165547	3.57726188	0.01392056
FASN	-0.1915223	2.68200527	-3.5754517	0.01394881
RIPK4	-0.2334931	3.61686223	-3.5727668	0.01399082
LINC00240	1.14775529	0.45070167	3.57074108	0.01402261
ERICH1	0.19472136	2.02840967	3.56634653	0.01409187
TTYH2	0.18268758	1.10403896	3.55655677	0.01424753

LINC00242	-1.1540416	-1.0050426	-3.5546843	0.01427752
SLC25A1	-0.1009233	3.56301769	-3.5537522	0.01429248
CALML6	0.61995899	1.55254892	3.55333523	0.01429917
PRR4	-2.9031168	6.77854258	-3.5410137	0.01449866
NR0B1	-1.1835639	-1.4920771	-3.5403209	0.01450997
DDX59	0.1460993	2.43078478	3.53460514	0.01460365
CA4	-1.6536121	-1.2566332	-3.5318027	0.01464982
USP35	-0.1675148	0.581222	-3.5263884	0.01473951
PCDHAC2	-0.4201677	0.20051318	-3.5256088	0.01475247
A2ML1	-0.7698287	-1.6995896	-3.5090772	0.01503042
GSTCD	-0.1377995	0.80695952	-3.5004532	0.01517774
ZNF263	0.11845121	2.52221303	3.49890105	0.01520443
DHFRL1	-0.1554194	1.72829888	-3.4885235	0.01538422
CSRP3	-0.5814727	-2.0118487	-3.4844607	0.01545526
MCM8-AS1	-0.8182792	-1.7911285	-3.4810913	0.01551445
BHLHA15	-1.9045772	-0.3396511	-3.4794048	0.01554418
NDUFA4L2	0.8412857	2.25504362	3.47005489	0.01571012
OR2C1	-0.8637147	-1.6826203	-3.4622152	0.0158508
ACSL5	0.19253944	2.84679563	3.46202772	0.01585418
STAT5A	0.23075822	2.4745388	3.45939247	0.0159018
FLJ41941	-0.3086944	-2.1482379	-3.459275	0.01590392
TWISTNB	-0.1157861	1.76958985	-3.4563762	0.01595649
UBXN8	0.2690862	1.69485128	3.45224689	0.01603171
ZNF646	-0.0807846	1.70282242	-3.451557	0.01604432
YWHAB	0.08399471	4.53789053	3.44811805	0.01610733
GAS2L1P2	-0.6713966	-1.7242799	-3.445958	0.01614704
LOC101929331	-0.8874513	0.95023173	-3.4389989	0.01627575
FAM35A	0.20512723	2.25003103	3.42940246	0.01645512

FAM200B	0.19668765	1.63756683	3.42812684	0.01647913
SAA2	-0.7576724	3.83740898	-3.4280467	0.01648064
MRPL32	0.15109451	3.57022399	3.4265459	0.01650894
TBCE	0.24411407	1.98088277	3.41860939	0.0166595
MRAP2	-0.3132615	1.49117428	-3.4145038	0.016738
RNGTT	-0.2293628	1.0978822	-3.4135633	0.01675604
GUF1	0.04884268	2.22364923	3.40655997	0.01689105
PRB4	-4.9156829	4.05087856	-3.4058631	0.01690455
FNTA	0.06935508	3.53096832	3.40510388	0.01691927
GLUD2	-0.4629969	-0.646417	-3.3937857	0.01714046
ZBTB12	-0.3264276	1.62034308	-3.3930876	0.01715421
SNX20	0.71029162	0.27492578	3.39117968	0.01719185
BRD9	0.30741475	2.73254247	3.38163691	0.0173815
C1orf100	-1.2279316	-1.4915516	-3.3806419	0.0174014
AKR1C1	-0.4153387	4.35531369	-3.3681526	0.01765347
IL17REL	-0.8000489	-1.5595735	-3.3646108	0.0177257
DMAP1	0.11008779	3.3487957	3.35585717	0.01790563
ZNF138	0.34167967	1.63357537	3.34940344	0.01803959
CC2D1B	-0.1440944	1.81738404	-3.3455915	0.01811924
DUS3L	0.14246288	2.7217419	3.34161829	0.01820268
FAAH2	-0.2732925	2.05953571	-3.3394385	0.01824864
EVI5L	-0.1516433	2.01654626	-3.3381518	0.01827583
SLC25A21-AS1	0.27594413	1.00257792	3.33662588	0.01830813
NGDN	0.18239436	3.29627889	3.33647476	0.01831133
HTR7P1	0.36775274	0.38482634	3.33477085	0.01834749
PIK3R4	-0.1882233	1.86928748	-3.3340117	0.01836362
SIX1	0.07353356	3.44067479	3.32442291	0.01856877
ASCC3	-0.1334357	1.85395872	-3.3207769	0.01864745

DDX31	0.12366374	1.35700618	3.31269633	0.01882315
LGALS8-AS1	0.65054282	-0.4253843	3.31180337	0.01884268
NARS2	-0.1923373	1.99297221	-3.3092885	0.0188978
MMS22L	-0.3385668	0.24920082	-3.2969806	0.01917018
GMPR2	0.13964575	3.82535581	3.29417188	0.01923295
FOXN3-AS2	-0.5901409	-1.8590566	-3.2931306	0.01925628
ENO3	0.54272251	0.72189347	3.29190325	0.01928382
GRM2	0.71133572	-0.6265914	3.29004663	0.01932556
PDZK1IP1	-0.3685329	3.13660007	-3.2894945	0.01933799
PRR14	0.09242338	3.02086729	3.2871863	0.01939007
LINC00691	-0.3176047	-2.1437828	-3.2862907	0.01941031
RAB40B	0.10942213	2.61217943	3.28615404	0.0194134
SNX30	-0.2283395	0.89559495	-3.2803903	0.01954429
GCOM1	1.00689748	-1.2631091	3.27857307	0.01958576
PDCL3	-0.2113788	3.1178849	-3.2699086	0.01978484
DCANP1	-0.7512724	-1.5820695	-3.2691418	0.01980257
ARL2	0.13215515	4.20297623	3.25889795	0.0200411
ATG7	0.15204467	1.92474671	3.2568198	0.02008988
PIP	-1.9337083	4.55680693	-3.2493631	0.02026599
ZSCAN21	-0.3186662	1.79486098	-3.2489671	0.0202754
NAT8L	-0.5942026	-1.7929218	-3.2464075	0.02033628
POLR2H	0.1705747	3.54944912	3.24633367	0.02033804
SNIP1	-0.1147755	1.68637852	-3.241404	0.02045588
SLC2A1-AS1	-0.8538935	-0.5067565	-3.2406975	0.02047283
LOC101927604	-0.9935494	-1.5215309	-3.2384118	0.02052778
SMOC1	-0.9032783	-1.2500834	-3.2297293	0.020738
FOXD4L4	-0.5671279	-2.0190212	-3.226289	0.02082196
SEC11A	0.19772742	4.0951427	3.2251272	0.0208504

ESF1	-0.151763	1.89487371	-3.2176561	0.02103432
CFB	-0.3298761	4.42729379	-3.215563	0.02108617
THRB	-0.2571699	1.28736324	-3.2147769	0.02110568
DPY19L1P1	0.44586286	-0.2498431	3.21322414	0.02114427
XXYLT1-AS2	-1.4840345	-1.108044	-3.2118743	0.02117789
MAP1LC3B2	0.46469963	0.12879197	3.20843111	0.02126391
GAPT	0.73939841	-0.4285273	3.2074691	0.02128801
MYRFL	-0.6928782	-1.8365619	-3.2055825	0.02133536
SEZ6	-0.2537127	-2.1757288	-3.2010294	0.02145013
PNP	0.30045458	2.2406167	3.19473716	0.02160987
LOC100289473	-1.1611821	-1.5430468	-3.1947119	0.02161052
SAMD14	1.16128853	-0.6447831	3.18779128	0.02178774
EVA1B	0.24001718	3.39652386	3.18345761	0.02189954
ZBTB6	0.34434836	1.5122469	3.18196094	0.0219383
ZNF234	0.2224766	1.19476337	3.17948478	0.0220026
HES7	-0.779459	-1.8291015	-3.1745377	0.02213167
ILDR2	-0.559365	-1.6793406	-3.167557	0.02231525
AVPI1	-0.5586208	4.00017732	-3.1640081	0.02240922
LOC102467147	-1.2930631	-1.3979648	-3.1576028	0.02257994
COPS7B	0.16460763	2.71249497	3.14869945	0.02281965
CRISP3	-2.3409196	1.84011814	-3.1469624	0.02286675
BMF	0.23270291	1.51293197	3.14241125	0.02299066
IKBKB	0.15085947	3.01793774	3.14200478	0.02300176
DRP2	-0.2837542	-1.9536989	-3.1406169	0.02303971
HIST2H4A	-1.0172851	-0.9890468	-3.1307503	0.02331154
HPGDS	0.70504076	0.12590491	3.12850019	0.02337403
GTF2IRD2	-0.1451414	1.33178953	-3.1271945	0.02341038
NLRP12	1.03018007	-1.4491481	3.12684151	0.02342021

LY96	0.73149157	1.52853386	3.11955428	0.02362432
LOC100506474	-0.8465772	-1.6568672	-3.1174364	0.023684
TMEM215	0.53972639	-1.3247792	3.11512421	0.02374936
PARP2	0.20800933	2.62116168	3.11491327	0.02375533
SLC9B2	0.54625408	0.71011296	3.11374919	0.02378832
KHDC1	-0.2890255	0.96613175	-3.1135695	0.02379341
TMEM220	0.1558317	2.20978777	3.11298107	0.02381011
PCDHGB1	-0.9174672	-1.2857453	-3.108117	0.02394864
RNVU1-14	-2.7934013	-0.9058845	-3.1054673	0.02402448
SNAPC3	0.15022162	2.31799448	3.10357879	0.02407869
DNM1P46	-0.6607968	-1.5781041	-3.1021377	0.02412016
PHF20	-0.0909983	2.21081291	-3.0998706	0.02418554
LOC100505841	-1.7519218	-0.1173801	-3.0986663	0.02422036
GALR3	-0.6705662	-1.967302	-3.0982684	0.02423187
ATP1B2	0.72119039	0.28895564	3.09605794	0.02429595
SPATA5L1	-0.1127464	1.49238214	-3.0907775	0.02444978
HIST1H2BN	-1.4983062	-1.1997681	-3.0906244	0.02445426
TMEM140	0.18605938	2.37409716	3.08941667	0.0244896
SEPHS1	0.11544073	2.99120214	3.08407749	0.02464653
LINC00235	-1.6569777	-1.4740962	-3.0733353	0.02496564
LAMTOR1	0.13570838	3.87934201	3.07226637	0.02499764
PAGE5	-1.1935615	-1.7058043	-3.0638376	0.02525159
EGLN2	0.12614902	4.04100955	3.06151184	0.02532217
HECW1	0.51964074	-1.377281	3.06077713	0.02534451
NEBL-AS1	1.04487843	1.59411013	3.0600832	0.02536563
C1QL1	-0.7271673	-1.5005203	-3.0573606	0.02544868
LOC102723809	-1.3390737	-0.9521638	-3.0568533	0.02546418
SNORA63	-3.8908087	-0.3571808	-3.0495044	0.02569001

LOC100505912	-0.8773213	-1.6175486	-3.0484407	0.02572288
PKD1L3	-0.3361108	-2.0356685	-3.0477899	0.02574302
MED8	-0.1379984	2.33547227	-3.0477382	0.02574462
ANP32AP1	0.81419476	-1.659823	3.04498151	0.0258301
EEF1E1	-0.3339531	2.26195502	-3.0438675	0.02586473
SNX4	-0.2201715	2.53400248	-3.0435364	0.02587504
ARHGAP24	0.16202526	2.28014489	3.04201092	0.02592257
KRTAP5-1	-1.0059627	-1.315336	-3.0396373	0.02599672
TAC3	-1.13335	-1.7359101	-3.0396127	0.02599749
ZNF7	0.15690986	2.50422057	3.03855377	0.02603064
FCGRT	0.17175012	4.65026686	3.03453649	0.02615685
HIST1H4C	-1.8098486	-1.3976608	-3.0345004	0.02615799
EMC3	-0.1446293	3.81422002	-3.0341191	0.02617001
PHF10	-0.0817116	2.94842622	-3.0337231	0.02618249
FAAP100	0.13427064	2.40949682	3.03272453	0.026214
NDEL1	-0.100133	3.09977588	-3.030414	0.02628707
CCDC183	0.69046592	0.02865184	3.0291993	0.02632558
TM4SF1	0.41259864	4.09168082	3.02843518	0.02634983
SP110	0.11989571	2.04991415	3.02774361	0.02637181
MPHOSPH6	-0.1911168	2.65910781	-3.0246845	0.02646924
PPP2R5E	-0.1169218	2.29413379	-3.0223608	0.02654352
ARHGAP11B	-0.6894713	-1.5280589	-3.0169629	0.02671696
EPHA1-AS1	-0.3895901	-2.0549932	-3.0143709	0.02680069
FAM181A	-0.4701556	0.77617821	-3.011058	0.02690813
TPSG1	1.16427622	-1.1199914	3.01022288	0.02693529
MPPED2	0.40609469	0.97318952	3.00928444	0.02696584
FAM74A7	-0.6295636	-1.8109089	-3.0084808	0.02699204
HAND2	-0.7492354	-1.4204736	-3.0065999	0.02705346

SMA5	-0.4100435	-2.0179372	-3.006354	0.0270615
C16orf86	0.41917582	1.08915029	3.00502119	0.02710513
MAP4K4	0.23199749	2.63646793	3.00388117	0.02714251
SH2B1	0.07347628	3.44973236	2.99920372	0.02729648
RHBDL1	0.43425364	1.69884162	2.99883702	0.02730859
CCDC117	0.10303497	2.24451278	2.9985296	0.02731874
ZNF815P	0.42230849	-0.3947599	2.99819331	0.02732986
FAS-AS1	-0.5979617	-2.0036043	-2.9964914	0.0273862
CCDC154	1.02160631	-0.5461428	2.99275698	0.02751026
MYLK4	0.38750458	-0.1006292	2.98846468	0.02765363
HIST1H2BG	-1.8209641	-0.308689	-2.9856811	0.02774704
LDLR	-0.4097088	3.23152099	-2.983915	0.02780649
E2F2	0.4048887	-0.9641242	2.98281525	0.02784358
AP1S1	0.18916481	3.39095357	2.98147209	0.02788895
MMP15	0.2040827	2.21082194	2.97692106	0.0280433
TCEA2	0.14598629	2.91347303	2.97664818	0.02805258
NCRNA00250	-1.2545303	-1.6753199	-2.9758576	0.0280795
POT1-AS1	0.57486765	-1.2764901	2.97461817	0.02812176
LYZ	-1.9189026	6.56360913	-2.9716319	0.02822386
ANKRD61	-0.85365	-1.3851866	-2.9682073	0.02834145
PCSK1	-0.4578427	-1.7120612	-2.9674611	0.02836715
CFAP58-AS1	1.43912086	-0.8899749	2.96651148	0.02839988
SLC22A1	-0.7808528	-1.2613965	-2.9657742	0.02842533
PCDHB13	-0.3248116	0.82092798	-2.9653955	0.02843841
PLOD3	0.23041842	2.45023933	2.964967	0.02845321
CSNK2A3	-0.9048562	-0.7813447	-2.9627711	0.02852923
TRMT1	0.17102238	2.56212833	2.96122484	0.02858289
TERC	-1.7900199	-1.0350822	-2.955142	0.02879507

RNF39	-0.3941008	2.00938359	-2.9546446	0.02881249
KDR	0.53394938	2.05043911	2.95295861	0.02887165
RFT1	0.24790688	1.38263818	2.95263598	0.02888298
LRRC70	0.66134718	0.09377732	2.95083844	0.02894623
ERC2	-0.4518758	-1.9606433	-2.9434149	0.02920903
POLE4	0.31346664	3.63148239	2.94335807	0.02921106
LOC400927-CSNK1E	1.38769643	0.08798222	2.94220215	0.02925222
DEAF1	0.11203049	2.62095971	2.94140594	0.0292806
HIST1H2AM	-1.2977132	-1.6537285	-2.939483	0.02934929
CFLAR-AS1	-0.9496282	-1.827771	-2.9375622	0.02941808
AMHR2	-0.8036012	-1.8324101	-2.9319657	0.02961952
FDXACB1	0.27581019	0.62342111	2.92722987	0.02979116
EXOSC6	-0.0997231	3.06491635	-2.926203	0.02982853
COL10A1	0.87147354	-0.4544817	2.92552388	0.02985326
LOC100506406	-1.3395924	-1.6327889	-2.924364	0.02989557
DHCR7	-0.1429031	2.63268433	-2.9233548	0.02993243
PCDH7	-0.3464115	2.47706018	-2.9211592	0.03001279
AOC1	0.80412497	-0.473894	2.9126187	0.03032766
RHBDL2	-0.2018539	2.27554798	-2.9126158	0.03032777
SND1-IT1	0.96299671	-1.3553706	2.90545605	0.03059453
LINC00574	-0.3010422	-2.0513136	-2.9001545	0.03079372
AVPR1A	0.99449642	-0.8692696	2.89999115	0.03079989
INSIG2	0.14120487	2.03569198	2.8994732	0.03081943
LOC101927623	-0.3596966	-2.1227368	-2.8976884	0.03088687
C10orf10	0.66442291	2.48785404	2.89758893	0.03089063
LOC101927571	-1.2132895	-1.6959404	-2.8960355	0.03094947
NECAB2	-0.3454404	-2.0430219	-2.8904599	0.03116167

SNAPC2	0.27535801	2.68459091	2.8899856	0.03117979
PCAT29	-1.3305477	-1.4734524	-2.8891786	0.03121066
LOC101929412	-0.606404	-1.9993831	-2.8875376	0.03127353
SORD	0.13018136	3.36035657	2.88328192	0.03143721
OR1L8	-0.678612	-1.9632791	-2.881911	0.03149014
SH2D5	-0.3502816	-2.0055922	-2.8794197	0.03158657
HOXD1	0.65714022	-0.6111633	2.87785311	0.03164738
MUC13	-0.5461002	3.08897304	-2.8758208	0.03172645
PIGZ	0.27427129	1.43491228	2.87451958	0.0317772
PPP1R35	-0.2324331	3.11344023	-2.8719497	0.03187768
KCTD13	-0.1372956	2.54032209	-2.8713723	0.0319003
LOC102503427	-1.4010496	-0.5875531	-2.8693611	0.03197924
FOXD3-AS1	-1.3770342	-1.614068	-2.8681092	0.03202848
SCGN	-1.1373504	-1.1008508	-2.8678328	0.03203937
SRPX2	-0.3889966	2.09744716	-2.8674885	0.03205293
LOC646626	-1.2891604	-1.07908	-2.8671404	0.03206665
PHEX-AS1	-0.6514938	-1.9768382	-2.8664413	0.03209422
TCFL5	0.16160522	2.10931448	2.86521793	0.03214253
ACE	0.49203508	2.56002134	2.86424688	0.03218094
MYCN	0.64510255	-0.5126435	2.86256726	0.03224748
IFIT1	0.22610104	1.51189151	2.86226692	0.0322594
HNRNPA3P1	-0.2470646	-2.1394638	-2.8550531	0.03254705
HBP1	0.12644433	3.3870907	2.85471633	0.03256054
SCNN1G	-0.5527524	2.88067669	-2.8545735	0.03256627
NR1D2	-0.2397247	3.02687862	-2.8516106	0.0326853
RPA2	0.12753004	3.21682351	2.85158727	0.03268624
SLC25A25	-0.4456605	2.95959544	-2.8515483	0.03268782
SYT4	-0.1687671	-2.2182015	-2.8496817	0.03276306

RPL34-AS1	-0.4719977	-2.0267205	-2.8475349	0.03284983
RIPPLY3	-1.0089836	-1.4253265	-2.8439164	0.03299665
KCNK10	-0.1162753	-2.2444474	-2.8431357	0.03302843
HIPK1-AS1	0.83165219	0.02364391	2.84297268	0.03303507
RPS14P3	0.5546816	0.49267784	2.84273832	0.03304461
MYBBP1A	-0.1231525	2.669681	-2.8384123	0.03322137
ACER1	-0.720756	-1.9422071	-2.8338595	0.03340852
TMEM255A	0.68220054	-1.4041627	2.83177213	0.03349471
HLX-AS1	-1.5335336	-1.5358183	-2.8316474	0.03349987
MAPK12	0.5571348	0.9858301	2.83090308	0.03353067
LOC101926911	-0.8396187	-1.4865423	-2.8291539	0.03360316
PPP2R2C	-0.5914112	-1.8453812	-2.8286644	0.03362348
ZNF705E	-0.4870497	-1.9165006	-2.8247877	0.03378488
RNF167	0.09413594	4.24434019	2.82400932	0.03381738
UBAP2L	-0.0587454	3.86314519	-2.8227226	0.0338712
LOC388406	-0.7326353	-1.9362675	-2.8200187	0.03398458
INF2	0.15138665	2.60765723	2.81526687	0.03418486
OTUD5	0.06733852	3.41755282	2.81458935	0.03421352
LINC00412	-0.864562	-1.4653097	-2.813248	0.03427034
ARHGEF7-AS2	-0.4574535	-2.0738584	-2.8113275	0.03435187
LINC00992	-0.3399045	-2.0135512	-2.8110897	0.03436198
SCNN1D	0.41483706	1.21329281	2.80789741	0.03449802
ZNF350-AS1	-0.7640434	-1.9205634	-2.8076016	0.03451065
WASH7P	0.40588536	1.47565281	2.79880331	0.03488879
LINC00870	-0.2926757	-2.1562472	-2.7942627	0.03508571
BHLHB9	0.31738261	0.88401771	2.79267692	0.03515477
SRMS	-0.4460804	-0.5317204	-2.7896481	0.03528709
DPP6	-0.099226	-2.2529721	-2.788959	0.03531727

LINC01138	-0.4743177	1.03443758	-2.7887325	0.03532719
GCSAM	-0.4824365	0.10568248	-2.7801072	0.03570746
VAX2	-0.5275396	-2.0388153	-2.7791561	0.03574966
TRAIP	0.35423352	0.29443186	2.77670726	0.03585858
ALG5	0.16394783	3.02853006	2.77470762	0.03594779
CEP120	-0.1129396	2.02453004	-2.7736231	0.03599627
SLC5A2	-0.8373529	-1.7540814	-2.7723223	0.03605451
CCDC7	0.4468773	0.1133088	2.77211854	0.03606365
PIN4P1	-0.3186406	-2.1432648	-2.7713897	0.03609634
HTR4	0.38247716	-2.1113465	2.77062755	0.03613055
GABRA2	-0.3890724	-2.0180041	-2.7654833	0.03636244
NAGS	0.65147847	1.00594705	2.76521704	0.03637449
ZFP91	-0.1318154	2.89466489	-2.7648847	0.03638953
LOC101929023	1.44246769	-1.5813512	2.76337743	0.03645783
STMN1	0.17464846	3.11207344	2.76056791	0.03658553
LOC441454	0.86943283	-0.0614573	2.75853641	0.03667816
PPEF1	0.91342087	-1.2893157	2.75669033	0.03676257
PRB1	-4.7471653	1.84653232	-2.7549876	0.0368406
WDR89	0.20715031	1.45189237	2.75335669	0.03691552
CRAT37	-0.6270754	-1.5973951	-2.748319	0.03714797
MAP2K1	-0.1943008	2.80591291	-2.7482438	0.03715146
GDPD2	-0.4478795	-1.7364458	-2.747013	0.03720849
RAPGEF4	0.60818027	0.74395133	2.74590898	0.03725974
2-Sep	0.03428917	4.79274987	2.74560019	0.03727409
NTN3	-0.5404275	-1.67907	-2.7447779	0.03731233
PRB3	-3.5957609	4.43460702	-2.7446715	0.03731728
ADPRM	0.24495315	1.38151943	2.74426746	0.03733608
SSR2	0.18702192	4.77801615	2.7434147	0.03737581

LOC339666	-0.5267994	-2.0391854	-2.7424725	0.03741975
SLC46A3	-0.0691979	2.30187491	-2.7421619	0.03743425
DERL1	-0.1609553	3.02378513	-2.736332	0.03770753
PRR5	0.26205247	2.71137617	2.73315141	0.03785754
CHRM4	-0.5786277	-1.5493502	-2.7301259	0.03800084
LOC100130950	0.35873008	0.7578004	2.72976996	0.03801774
HTRA2	0.16436655	2.20704467	2.72443171	0.03827214
DGKB	-0.6729816	-1.7871213	-2.7205576	0.03845793
TBXA2R	0.85010869	0.62683997	2.71755131	0.03860278
CRSP8P	0.41741914	-2.0938755	2.71536646	0.03870842
TUBD1	0.27048303	1.56093205	2.71448937	0.03875092
SETMAR	0.15305843	2.31168705	2.7138479	0.03878203
PRSS36	0.5294737	0.47159408	2.71333649	0.03880686
SETDB2	0.20354167	1.56024407	2.71182609	0.03888028
PHF14	-0.1472409	2.40100462	-2.7110113	0.03891995
PLCB1	0.22801942	0.75647598	2.71031906	0.03895368
LOC100507661	-1.1117047	-1.0117325	-2.7102667	0.03895623
TFAP2E	0.28742761	0.32709574	2.70862511	0.03903637
FAM153C	0.55033258	-0.6235352	2.70661917	0.03913454
EHMT1-IT1	-0.5249401	-2.040115	-2.70415	0.03925574
PABPN1L	-0.5850779	-2.0100462	-2.7028662	0.03931892
CCL3L3	-1.223372	-1.3852603	-2.7024147	0.03934116
CTHRC1	0.94776201	1.41732323	2.70192493	0.03936531
KRTAP5-8	-0.6916087	-1.6352362	-2.7015203	0.03938527
CNTN4-AS1	-0.7260517	-1.7095682	-2.7011097	0.03940554
GIMAP5	0.7934521	2.37297626	2.70034746	0.03944319
RTEL1	0.25384487	1.52659968	2.69656724	0.03963051
TMEM191C	-1.0275742	-1.3370625	-2.6960002	0.0396587

CABP4	0.32141401	-0.7116839	2.69549183	0.03968398
MPHOSPH10	0.08350269	2.94772384	2.69291931	0.0398122
ABCG1	0.13703044	2.8010098	2.69064415	0.03992597
NAIF1	-0.1430525	1.26902176	-2.687202	0.04009876
CAMTA2	0.08618551	2.71446975	2.68714707	0.04010153
ANKRD20A11P	-0.7259176	-1.4907469	-2.6869284	0.04011254
CCL13	-1.0572104	-0.9493058	-2.6865384	0.04013217
TNNT1	0.92497764	-0.3164273	2.68639701	0.04013929
HCG25	-1.2019098	-1.5389243	-2.6862051	0.04014896
MAGEA5	-0.4490539	-2.0780581	-2.6861528	0.0401516
ASB11	-0.2049769	-2.2000967	-2.685735	0.04017266
LOC644656	0.44001386	0.77483551	2.68460981	0.04022943
CYSRT1	-0.5635262	1.10471473	-2.6835059	0.04028522
UBR5-AS1	0.52885073	0.79731679	2.68287025	0.04031738
H19	1.19397958	2.41780085	2.68257328	0.04033241
RSRC1	-0.1656625	1.83463638	-2.6792451	0.04050133
KIF25-AS1	-0.6191514	-1.9930094	-2.6771157	0.0406098
CYP51A1-AS1	-0.7127329	-1.4012753	-2.675329	0.04070107
LOC100506688	-0.4076955	-1.895888	-2.6750843	0.04071358
KCNAB3	0.43200852	0.43269016	2.67374737	0.04078204
COLCA2	0.21470314	3.00277042	2.6735788	0.04079068
MAGEL2	0.65604888	-1.256572	2.67256631	0.04084262
CCL14	0.9625753	4.72721319	2.67201832	0.04087076
PLA2G6	0.23502483	2.85425504	2.67181762	0.04088107
NDUFAF1	0.30046237	2.53823925	2.67084611	0.04093103
HMGB1	0.09671673	4.5341386	2.66721467	0.04111834
ACYP2	-0.2914566	2.29802998	-2.666728	0.04114352
RASA4	0.58462592	1.27726365	2.66620549	0.04117056

CXCL6	-0.5123302	2.66760889	-2.6650957	0.04122807
15-Sep	0.13520199	4.20735962	2.66408001	0.04128078
BDKRB2	-0.1770687	1.87504053	-2.6625722	0.04135917
CYP26C1	-0.76775	-1.5273002	-2.6616887	0.04140517
IGF2	0.87057635	2.25336626	2.66072222	0.04145556
LOC101928304	-0.8480912	-1.8785395	-2.6590291	0.04154399
UBR2	-0.1094525	2.16795863	-2.6578945	0.04160337
DACT3-AS1	-1.0181417	-1.3900203	-2.6568386	0.04165871
ARHGAP26-AS1	-0.4696361	-2.0677671	-2.6546339	0.04177452
HSCB	0.23000618	2.33412455	2.65428537	0.04179286
PIK3C3	-0.1136227	2.16477879	-2.6531951	0.04185028
FUT7	-0.5682636	-1.8348738	-2.6518139	0.04192315
LINC01569	-0.1858926	0.53542881	-2.6506704	0.04198359
TM4SF18	0.72933343	1.05086342	2.64882574	0.04208128
ATP5E	0.18481464	5.86436281	2.64759302	0.0421467
PPM1N	0.24583898	0.00709729	2.64678479	0.04218965
LINC00272	-0.4639017	-2.0706342	-2.6410344	0.04249662
SESN2	-0.2565026	1.74809868	-2.6388267	0.04261512
SPRR2A	-1.3895772	-1.6077965	-2.638501	0.04263263
PAH	-0.2926503	-2.1562599	-2.6355626	0.04279097
LRRK1	-0.1138446	1.84230663	-2.6352159	0.0428097
CSNK1G2-AS1	-0.8583872	-1.8733915	-2.6329484	0.04293238
INSL3	-1.1539547	-0.7550621	-2.6305588	0.04306208
FDPSP2	-0.667905	-1.3394041	-2.6277048	0.04321755
PDCD6IPP2	-0.7842237	-1.382435	-2.6262814	0.04329531
C11orf91	-1.0594043	-0.6121907	-2.6246701	0.04338352
MYADML2	-0.3287124	-2.1382289	-2.6246275	0.04338585
ZNF181	0.18786678	1.801236	2.62318415	0.04346504

SFTPB	0.79730792	-1.2188407	2.62254687	0.04350005
SS18L2	0.2072483	2.79523082	2.622474	0.04350406
BRD8	0.11554654	2.84329212	2.62183542	0.04353918
SLC26A9	-0.9616676	-0.9858933	-2.6212287	0.04357257
FATE1	-0.7707433	-0.1091121	-2.6210011	0.04358511
C3	-0.5124865	4.76544079	-2.6207683	0.04359794
PDE10A	0.55394066	-0.4313764	2.61994775	0.04364317
TOX3	-0.2701073	2.32221597	-2.6199295	0.04364418
HMG20A	0.14539351	2.28031634	2.61939353	0.04367375
HTR3B	-0.2776801	-2.1637451	-2.6193002	0.0436789
SLC26A4	-1.125079	0.14923283	-2.6181913	0.04374017
RAB42	1.22221256	-0.0585131	2.61816164	0.04374181
LOC101929524	-0.3750384	-2.1150659	-2.6180477	0.04374811
MANEAL	0.33079521	0.98868172	2.61802856	0.04374917
RGL2	0.20712752	3.58039671	2.61653366	0.04383194
PILRB	0.42607589	2.80204237	2.61621416	0.04384965
JKAMP	-0.1545886	2.90435394	-2.6161696	0.04385212
NABP2	-0.1086754	2.89948486	-2.6140425	0.04397023
AHCYL2	-0.1618533	2.67773313	-2.6137158	0.0439884
UBQLN2	-0.1491247	2.85724741	-2.6128341	0.04403748
GGN	0.67636386	-0.5722825	2.61183733	0.04409304
GAS2L3	0.23725493	-0.8856063	2.61160347	0.04410609
CD99	0.24374567	5.32048761	2.60978987	0.0442074
SLC46A2	-0.7411323	-1.5023567	-2.6094042	0.04422898
YTHDF1	0.0860344	3.03027184	2.60863452	0.04427208
LOC100287592	-1.630243	-1.2024507	-2.6083896	0.0442858
CCDC102B	0.95679609	-0.2128193	2.60621227	0.044408
KCNK5	0.34788677	1.86804793	2.60614391	0.04441184

ATP4B	-0.5265268	-2.0393217	-2.6060123	0.04441924
LINC01208	-0.5695503	-2.0178099	-2.6048669	0.04448369
HCN2	-0.4878118	-1.7566228	-2.6046344	0.04449678
PTCHD2	-0.5566848	-1.932885	-2.6028599	0.04459686
CSNK1G3	0.10011628	2.12905477	2.60267253	0.04460744
VAMP4	0.15679666	1.76328111	2.60043418	0.04473406
TMEM92-AS1	-1.1687433	-1.7182134	-2.5997028	0.04477552
ZNF410	0.10511464	2.58463683	2.59904512	0.04481283
ZNF490	0.24716322	1.11359466	2.59747582	0.04490201
ZNRF3-AS1	-0.2987107	-2.1532297	-2.5963805	0.04496436
VAV1	-0.1472789	2.57738524	-2.5949126	0.04504807
RPS6KA5	-0.3467545	1.57098831	-2.5936955	0.04511761
DOCK7	-0.2214323	1.43342002	-2.5935407	0.04512646
CORO2A	-0.1531709	2.97774451	-2.5935337	0.04512686
CEBPD	-0.3304743	5.33045364	-2.5919288	0.04521875
GIMAP8	0.74311543	1.5091176	2.59136243	0.04525122
MYOZ2	-1.0610256	-1.7720723	-2.590118	0.04532267
DDN	-0.2268119	-2.1891791	-2.5899714	0.04533109
ZFP69	0.27036226	0.99481041	2.58984527	0.04533834
CTLA4	-1.0857144	-0.9470265	-2.5897385	0.04534448
GNE	-0.2340752	2.65954759	-2.5894698	0.04535993
NEK2	-0.7950452	0.24279906	-2.5886949	0.04540452
LMCD1-AS1	-0.5957434	-1.5351078	-2.5879545	0.04544717
SEC24D	-0.1264929	2.38643233	-2.5878268	0.04545453
FAM163A	-0.3392156	-2.1329773	-2.5869186	0.04550691
INPP4B	-0.177184	1.29903913	-2.5863296	0.04554092
GATB	-0.1188242	1.79769088	-2.5861191	0.04555308
GALNT9	-0.4143044	-2.0954329	-2.5851432	0.0456095

RNF180	-0.3736579	0.92989477	-2.5842761	0.0456597
STARD4-AS1	0.54621426	-0.7667036	2.58345989	0.045707
MAPK8IP2	-0.6380951	0.4717651	-2.5814565	0.04582333
SCEL	-0.9247803	-1.623979	-2.5787754	0.04597952
NTN1	-0.3302487	3.66791278	-2.5780257	0.04602329
SMARCE1	0.11871659	3.94236952	2.57697946	0.04608446
JPX	0.43183945	0.63346725	2.57208374	0.04637184
LINC01604	0.60981399	2.18987877	2.5715187	0.04640514
IGSF11	-0.4236529	-0.1299682	-2.5695286	0.0465226
SUCLG1	0.14191217	3.52216391	2.56919788	0.04654215
LINC00476	-0.3872468	1.35908481	-2.5668404	0.04668178
NBPF11	0.1593428	1.28700208	2.56635124	0.04671081
MCCC1	0.16344944	2.86377679	2.56603497	0.04672959
ACSL3	-0.1790604	3.14348917	-2.5643184	0.04683165
NEK6	0.16342994	2.74916009	2.56222542	0.04695643
TSC22D3	-0.2264436	4.35102306	-2.5580682	0.04720532
PAPD5	-0.2052401	1.04789347	-2.5574537	0.04724223
LOC100652768	-0.3795148	1.05044313	-2.5562334	0.04731563
PRC1-AS1	-0.7111907	-1.8373207	-2.5531639	0.04750078
TRIM50	-0.2722122	-2.166479	-2.5513477	0.0476107
PTMA	0.10525304	7.09291799	2.55114402	0.04762305
CHRNB2	0.61774492	-1.5535808	2.55042592	0.0476666
C5orf38	0.38678445	2.69671959	2.54982311	0.04770319
ZBTB8OS	0.18243285	2.26732854	2.54933158	0.04773304
LINC01280	-0.3661923	-2.119489	-2.5492481	0.04773812
LOC102723517	1.34512538	-0.4816171	2.5489606	0.04775559
SPC24	-1.3750494	-1.2122613	-2.5488966	0.04775948
RAD17	0.08812273	2.50918267	2.54845305	0.04778646

ABCD2	-0.5084121	-0.4152054	-2.5479245	0.04781863
NCK1-AS1	0.1937451	1.128944	2.54694181	0.0478785
HLX	0.68314571	1.04734535	2.54328337	0.0481021
DHRS12	0.17501334	2.55847984	2.5423563	0.04815894
LARP1	-0.0978642	3.57931964	-2.5415241	0.04821002
PRAP1	-1.0588733	-1.4191439	-2.5404923	0.04827344
LRFN5	-0.4343686	-1.8801828	-2.5402617	0.04828763
PHOX2B	-0.2588988	-2.1731357	-2.5398893	0.04831055
C2	0.43855452	1.75339504	2.53793693	0.04843089
BCL10	-0.1576046	2.12420091	-2.5373484	0.04846723
TMEM238	-0.2826249	3.17922839	-2.534616	0.04863634
BCL2L2-PABPN1	0.12917864	3.90329636	2.53408032	0.04866957
ADAT2	0.20287986	0.40662917	2.53260347	0.0487613
TDG	0.08275101	1.9712807	2.53211613	0.04879161
FAM86EP	0.43965515	0.81921689	2.53063381	0.04888394
CPEB1	-0.4060939	0.8596367	-2.5305704	0.04888789
DCAF4L1	0.4488435	-1.0448125	2.52823622	0.04903366
GSG2	-1.0191141	-1.0167613	-2.5265404	0.04913986
KRT4	-0.6457104	3.0655903	-2.5258788	0.04918136
SLCO2A1	0.65398126	2.69122801	2.5247915	0.04924964
ZNF71	-0.389143	0.96113296	-2.5238359	0.04930974
BBOX1-AS1	-2.0701815	-1.2674943	-2.521814	0.04943715
FIGNL2	0.51438768	-0.4816415	2.52173978	0.04944184
CCL20	-1.916999	-0.0843184	-2.5207628	0.04950354
NR2F6	-0.1211309	4.07361214	-2.5201293	0.0495436
TLX1NB	-0.9668094	-1.6011709	-2.5188338	0.04962561
LINC00504	-0.4475245	-2.0552908	-2.5175793	0.04970516
LOC101928259	-0.7404345	-1.9323678	-2.5173143	0.04972199

SCGB3A2	-2.5366421	-0.3289223	-2.5146844	0.04988928
SRP68	-0.0991162	3.37490887	-2.5138421	0.04994299
CAMK2B	-1.3477688	-0.5985847	-2.5135058	0.04996445
LINC00461	0.46749375	-2.0688382	2.51316046	0.0499865
KCNK2	-0.7891073	0.00787744	-2.5123624	0.05003749
KCNIP2-AS1	-0.8976491	-1.5944115	-2.5122738	0.05004315
APOL4	0.40900971	0.80745179	2.50966247	0.05021042

Supplemental Table 9B. Differentially expressed genes between pre and post placebo.

Gene name	logFC	AveExpr	t-Statistics	P.Value
PPP2R3B	0.27205861	2.33048993	12.9644527	2.78E-05
LCNL1	-1.7817221	-1.3342879	-11.334849	5.63E-05
PAK7	-0.6976777	-0.6546059	-8.575895	0.0002378
UQCC3	0.17277214	2.32136261	8.40775763	0.00026299
INPP5B	0.17516212	2.07796948	8.35213025	0.000272
KIAA0922	-0.1603407	1.55058409	-8.0649718	0.00032473
LINC00608	1.39192479	-1.6066227	7.22445999	0.00056382
FAM136A	0.18413284	3.14444225	6.79032673	0.00076605
HM13-AS1	2.55775604	-0.3462289	6.72303917	0.00080448
SEC14L6	-1.1736714	-1.4931715	-6.6536448	0.00084649
SNHG10	0.3762406	2.11365555	6.57444431	0.00089759
POLR2G	0.25434234	3.70305205	6.37365966	0.00104412
PIAS1	-0.3059783	2.61662397	-6.3234731	0.00108499
ZNF614	-0.3756634	0.65753833	-6.3230982	0.0010853
KLF9	-0.7972108	2.21881101	-6.230512	0.0011657
PRRC2B	-0.1537792	3.37710551	-6.1643618	0.00122741
HERPUD1	-0.3647555	4.20806898	-6.1505871	0.00124073
TSC22D3	-0.5291474	4.30431929	-6.0164968	0.00137959
SEC24B	-0.1763702	2.52333819	-6.0070956	0.00138999
CDS2	-0.2192253	2.12240462	-5.8861312	0.00153221
B3GAT1	-1.453832	-1.2044741	-5.8511016	0.0015765
HIST1H4C	2.13492996	-1.2351201	5.84530336	0.00158397
POLR2H	0.44152571	3.64947821	5.82177701	0.00161472
LOC283038	1.25174854	-0.9993475	5.79127374	0.00165563
TMEM54	0.41893425	3.72332945	5.78025854	0.0016707

MFF	0.18006365	3.14781852	5.7781657	0.00167358
PTK6	0.30577619	2.40469842	5.69882379	0.00178715
TNN	-1.1949844	-1.3620848	-5.6090993	0.00192652
PRKCA	-0.5768683	1.40764928	-5.571098	0.00198931
ADCYAP1R1	-0.8080205	-1.6040846	-5.5404302	0.00204172
AURKAIP1	0.42947865	4.62551293	5.48203372	0.00214601
NIPBL	-0.1855563	2.43220876	-5.4807446	0.00214838
ZNF382	0.68809205	0.2016173	5.46144205	0.00218424
FLJ31104	1.21529383	-0.9734003	5.45402595	0.0021982
AKR1C4	1.64436437	-0.9014719	5.43090835	0.00224239
ARPC4	0.1695341	4.40003659	5.22638184	0.00268154
LAMC3	-1.6364712	-0.9556486	-5.1599032	0.00284513
TBC1D13	-0.2861839	2.20036079	-5.1207092	0.00294698
ADCY9	-0.2295904	2.59046998	-5.1019009	0.00299734
TMEM132C	-1.4095423	-0.7675553	-5.0999056	0.00300274
CACNA1E	-0.3873029	-1.356092	-5.0890144	0.00303242
FRMPD4	-0.8726248	-1.6790465	-5.0045999	0.00327426
RASSF8	-0.6407778	1.49523898	-4.9914169	0.00331401
ACO1	-0.3692096	2.08350676	-4.9905667	0.00331659
RPL19P12	1.23565679	-1.2800581	4.99023095	0.00331761
ATP6V0A2	0.09030975	1.78855289	4.97834321	0.00335397
CPO	1.24005391	-1.2257801	4.95762781	0.00341844
SLC39A4	0.57207243	2.80249096	4.95490335	0.00342702
CFHR1	0.59816603	-1.2993773	4.94433237	0.00346057
LY86	-0.8142807	0.45689883	-4.9164656	0.00355082
NNT	-0.1972115	2.23175343	-4.8832763	0.00366186
HAL	1.35391541	-1.5966674	4.82178956	0.00387832
KIAA0430	-0.1992488	2.77490795	-4.8067624	0.00393344

PLEKHM3	-0.2446525	0.7101221	-4.8011031	0.00395443
MBOAT1	0.23877509	2.83491797	4.79462366	0.00397862
LOC101928841	-1.1501415	-1.5894888	-4.7944698	0.0039792
TBC1D3P1-DHX40P1	0.56703839	-1.892358	4.79402895	0.00398085
NDNF	-0.6437111	0.81437474	-4.7759575	0.00404927
POLR2J4	0.31173167	0.91382418	4.77430501	0.00405559
RNF152	-0.6857313	0.51844293	-4.724606	0.00425118
CSNK1E,LOC400927-CSNK1E	0.14224924	3.84008561	4.70164695	0.00434517
HYLS1	0.2266067	1.63898405	4.68459561	0.00441653
PCDHAC2	-0.665142	0.39855293	-4.6655743	0.00449772
NOC3L	-0.2441456	1.48748456	-4.6522607	0.00455557
RNF26	-0.1893113	2.63905716	-4.6435535	0.00459387
MANEAL	-0.4675493	0.56080009	-4.6197181	0.0047006
DERL2	0.1458474	2.28420348	4.59934901	0.00479408
SETD8	0.13450195	3.0555959	4.57064331	0.00492945
COMMD4	0.37634752	3.86787599	4.56792071	0.00494251
ASCC2	0.26423769	3.40155015	4.56685335	0.00494765
LOC283922	0.36726978	0.55847369	4.53580411	0.00509967
GFRA2	-1.2645334	-1.1810016	-4.5158674	0.00520011
TANGO6	-0.3029453	0.81335971	-4.5145544	0.0052068
GNGT2	-1.8317652	-1.0155642	-4.5105381	0.00522734
NCOA2	-0.3744588	1.51402936	-4.5058616	0.00525137
MRPL34	0.25159581	3.85205817	4.49824747	0.00529076
UTY	-0.2900405	1.47666283	-4.4827832	0.00537182
RNF180	-0.5715474	0.87734966	-4.4690325	0.00544509
CEP85	0.37558761	1.63452192	4.46413719	0.00547145
PHTF2	0.16103664	1.59848825	4.45685486	0.00551094

NCBP2-AS2	0.35805764	2.95771021	4.4442017	0.00558032
NDUFA11	0.06802855	4.63726819	4.44282991	0.0055879
HLA-DOA	-1.195592	1.40536552	-4.4403186	0.00560181
C19orf43	0.16398656	5.55960741	4.4239306	0.00569357
KCNQ1	-0.222099	3.09867847	-4.4110772	0.00576674
IL2RB	-0.4664822	1.95326052	-4.4065813	0.00579259
RPL23AP32	-2.3935295	-0.67802	-4.3984292	0.0058398
DENND4C	-0.2089467	2.07100558	-4.3913942	0.00588089
TMEM258	0.23818327	4.91561502	4.3674668	0.00602315
TSSC4	0.22169092	3.09721326	4.33190882	0.00624188
ATP6V0E2-AS1	0.70768104	-0.2365548	4.33119336	0.00624637
ABCA12	1.0635071	-0.4609173	4.32694019	0.00627316
CASC15	0.27449156	-0.1249102	4.31798671	0.00632998
HLA-DPB1	-0.6142395	3.10721614	-4.3033562	0.0064241
SAP30L	-0.290014	2.25883455	-4.276012	0.00660433
MRPL51	0.2743878	4.72935238	4.25924924	0.00671766
TBC1D10B	0.26745015	3.0631325	4.25444629	0.00675054
ARHGAP24	-0.2955489	2.0392507	-4.2428487	0.0068307
C17orf89	0.5046181	4.13617593	4.22877644	0.00692942
LINC00565	1.03097979	-1.1729855	4.22261271	0.00697318
TMEM38A	-0.414669	1.11809997	-4.2163355	0.00701806
GIMAP1-GIMAP5	-1.996726	0.0449359	-4.214782	0.00702922
EIF2A	-0.3001306	3.09396614	-4.2083516	0.00707562
MYEOV2	0.46766814	4.36315822	4.20348692	0.00711096
NSUN3	0.38608105	1.67225478	4.2025939	0.00711747
RGS1	-0.6590499	2.29368915	-4.2012836	0.00712703
DHX35	0.33808531	2.09615733	4.19663307	0.00716109
POPDC3	-1.2349111	-1.3882769	-4.1682768	0.00737281

LOC441155	-0.4011595	-1.4674414	-4.167888	0.00737576
SYT1	-0.9757357	-1.4127363	-4.1488907	0.00752166
LINC01578	0.31314574	3.66200105	4.14536563	0.00754909
CYP2G1P	1.32760743	-0.7728477	4.13903606	0.00759863
PPP1R35	0.39347407	3.17788731	4.11745055	0.0077704
PPP4R1L	0.3649425	0.01049593	4.11266563	0.00780907
CDRT4	0.23075871	2.72676838	4.10890619	0.00783961
MLST8	0.10710576	3.03210357	4.10213055	0.00789499
LSM7	0.38904846	4.42131685	4.08797118	0.00801216
HLA-DPA1	-0.6484592	4.33865905	-4.0791701	0.00808599
SPOCK3	-1.3801466	1.38069528	-4.0661843	0.00819634
NEK6	-0.2800673	2.32697328	-4.0484459	0.00834986
XPC	-0.1798145	3.10774744	-4.0477168	0.00835624
GTF3C5	0.16599324	3.32525793	4.02887838	0.00852301
TRPV2	-0.8774476	-0.200082	-4.0280374	0.00853054
RAD9A	0.37966125	3.01840534	4.02113156	0.00859269
LRRC58	-0.1880728	1.76542878	-4.0137244	0.00865991
FBXL17	-0.3461578	1.78330465	-4.0091398	0.00870182
TMEM255A	-1.4966876	-1.037832	-4.0090912	0.00870227
ARHGEF39	0.37513569	0.20228444	4.00488959	0.00874088
ISM1	-1.2033119	1.05921106	-4.0033666	0.00875493
KIAA0754	-0.4808733	0.84672686	-3.997338	0.00881077
ARSA	0.17979305	2.64535476	3.98908243	0.0088879
VLDLR	-0.4150869	2.0306323	-3.9867854	0.0089095
DOCK1	-0.271999	2.78788738	-3.9842921	0.00893301
ARV1	0.14253617	2.94626028	3.98354953	0.00894002
FBXO27	0.33515231	1.90958593	3.98195657	0.0089551
JCHAIN	-1.6694448	3.24859144	-3.9796705	0.00897677

MTDH	-0.1804312	2.71416306	-3.9766348	0.00900565
TGFBR3L	0.84331272	0.77331844	3.96851316	0.00908344
TMEM251	0.3503653	2.72478447	3.96414854	0.00912555
NPAT	-0.3683137	1.14654801	-3.962001	0.00914636
KCNH4	-0.7598474	-1.3671963	-3.9619852	0.00914651
STAG1	-0.3625775	2.01466789	-3.9548668	0.00921585
CADM3	-2.3613862	-0.0132252	-3.9542974	0.00922143
PPL	-0.3971872	4.05392204	-3.9383575	0.00937899
MRPL17	0.21913147	2.70783436	3.9380968	0.00938159
PRRC2A	-0.1845195	3.68239189	-3.9366861	0.00939569
ERCC2	0.21251229	1.99449131	3.9332265	0.00943036
MKRN2OS	0.70528144	2.02877647	3.92756794	0.00948738
C6orf89	-0.29938	2.93726708	-3.9206778	0.00955734
PTK2B	-0.1208315	3.08016528	-3.9159551	0.00960563
STT3B	-0.1614173	3.42189501	-3.9148468	0.009617
BCL2	-0.8880027	1.5819545	-3.9117463	0.00964889
AMIGO1	-0.6795337	1.06893118	-3.904091	0.00972816
SLC50A1	0.20871786	3.35716785	3.8945105	0.0098284
GAL3ST1	-1.4037614	-1.0733903	-3.8941856	0.00983182
WASF3	-0.8205435	0.77920256	-3.8883606	0.00989337
NDUFA13	0.17889611	5.73106965	3.88217973	0.00995915
EFTUD1P1	1.57964243	-0.8797224	3.87496664	0.01003655
CEP95	0.21679386	2.88477728	3.86624946	0.01013099
CDK12	-0.1009791	1.91249771	-3.8647502	0.01014734
UBE2J2	0.20644033	3.27183358	3.86121897	0.01018595
EGFR	-0.6698917	3.2420368	-3.859796	0.01020156
CCNI	-0.3119335	4.70388099	-3.8531909	0.01027436
FAM208B	-0.2137175	1.92963106	-3.8505243	0.01030392

PHF7	0.31713976	1.94557855	3.8415643	0.01040395
DESI2	0.18433027	2.24074255	3.83445575	0.01048409
DFFA	-0.3109485	2.22782552	-3.8327848	0.01050303
PPFIBP2	-0.3214257	2.63155377	-3.8310521	0.01052271
CXCR1	1.30112451	-1.2452233	3.82171186	0.01062952
JADE2	-0.2945324	2.57693488	-3.8188661	0.0106623
CPT1A	-0.2071218	2.97969743	-3.8156916	0.01069901
GPX3	-0.5394475	4.31395499	-3.8151595	0.01070518
MYO15A	0.51463681	-0.9290474	3.80640057	0.01080727
HLA-F	-0.2911444	3.8751778	-3.8060461	0.01081142
CHD6	-0.1539454	2.28736784	-3.7939456	0.01095434
DOLPP1	0.18931493	2.65025353	3.7869693	0.01103771
LATS1	-0.1237592	1.83657523	-3.7829699	0.01108583
SUV420H1	-0.1483907	2.59862392	-3.7749145	0.01118347
LSM1	0.37032312	3.28249538	3.77357686	0.01119977
ARHGAP35	-0.2584952	2.71719479	-3.7613192	0.01135046
GTF2I	-0.3224615	3.45106369	-3.7585318	0.01138505
TMEM184A	0.39867251	2.688131	3.75640064	0.01141157
LOC100506606	0.37906021	0.58474313	3.75317915	0.0114518
MGA	-0.1372653	1.5209491	-3.7388437	0.01163276
NCLN	0.09901145	3.08796862	3.72609663	0.01179638
ZNF395	-0.173218	3.3161805	-3.7215872	0.01185488
UQCRQ	0.34210144	3.7599953	3.71980269	0.01187813
LINC00265	0.33387279	2.02939293	3.71425605	0.0119507
CTDSPL	-0.315128	2.89124013	-3.7139263	0.01195503
H6PD	-0.2195615	3.02145886	-3.7133576	0.0119625
PTPRQ	-0.527451	-0.0811274	-3.7080887	0.01203198
CBY3	1.65728431	-0.4363803	3.70407509	0.01208521

H1FX	0.26628713	5.18181376	3.7031384	0.01209767
RNASEK	0.30630578	5.34770125	3.69172343	0.0122507
TRIM17	0.36716435	1.17629737	3.69089346	0.01226191
TMEM171	0.72835729	1.06211484	3.68977061	0.0122771
RGS19	0.48361025	1.95144261	3.68429959	0.0123514
DCAF15	0.33602106	2.96044788	3.68149253	0.01238972
PNPO	0.18724692	2.47950474	3.67343937	0.01250039
SLC2A5	1.03867846	-1.2332345	3.66976709	0.01255123
FIZ1	0.12777959	2.37163365	3.6675953	0.01258141
NPIPB4	0.37916248	-0.583772	3.66731562	0.0125853
HUWE1	-0.2037234	2.72079641	-3.6667124	0.0125937
MEMO1	0.4097785	2.20896394	3.66025307	0.01268403
LOC728730	0.53488037	0.96932038	3.65584266	0.01274613
SNORA70	-4.6347424	1.34945172	-3.6455446	0.01289246
TMEM44	0.43141546	2.50335611	3.64222551	0.01294003
DPYSL4	-1.2119404	-0.5788479	-3.6417838	0.01294637
SUGCT	-1.8618094	0.27881065	-3.6380094	0.01300073
HDGFRP2	0.14867996	3.63178581	3.62987004	0.01311884
ALKBH6	0.26033066	2.63727771	3.6290991	0.01313009
PRDX2	0.12084387	4.93441524	3.6255851	0.0131815
TMEM176A	-0.4392601	2.29768536	-3.6212824	0.01324477
TSKU	-0.3370578	3.69639744	-3.6175139	0.01330045
UGT2B7	1.09119141	-1.3524734	3.61601153	0.01332273
LRRC37A3	0.48443717	1.74057312	3.60442187	0.01349598
SH3GLB2	0.16941809	3.80834967	3.60223996	0.01352888
CCDC77	0.24369177	1.28741383	3.59882986	0.01358048
LTA	1.27331367	-1.2095842	3.59516389	0.0136362
TNNC2	2.41705446	-0.3447457	3.59043785	0.01370841

LINC01206	0.53382012	-1.0954633	3.58993673	0.01371609
DBF4B	0.43587619	1.00930598	3.58371299	0.0138119
USP43	0.44835406	2.499375	3.58096668	0.01385442
PEG3-AS1	1.36976853	-1.6177008	3.57520683	0.01394407
SLC28A3	0.3688637	0.35184129	3.574177	0.01396017
CIAPIN1	0.20921007	2.28486795	3.57240294	0.01398795
NAA38	0.31901428	3.63141834	3.57230444	0.01398949
BCL2L2-PABPN1	0.31971367	4.02182702	3.57183042	0.01399693
HARS2	0.30968382	2.57488869	3.57172566	0.01399857
FASTK	0.24220637	3.37337576	3.56912513	0.01403944
VEZF1	-0.1546876	3.23549169	-3.5689916	0.01404154
PSMB3	0.27433124	4.66031295	3.5673276	0.01406777
SYNE2	0.1554277	4.14051731	3.56702879	0.01407248
SORL1	-0.2584968	2.41537011	-3.5658303	0.01409141
LOC101928979	1.55610609	-0.3759542	3.56573998	0.01409284
C2orf68	0.18229292	2.36799331	3.55828326	0.01421128
TMEM238	0.33413049	3.33813823	3.55731211	0.01422679
LMAN1	-0.1991242	3.30987282	-3.5572587	0.01422764
CAPN12	0.45879223	1.59123986	3.55673551	0.014236
UBAP2	0.37353636	2.57944605	3.55671579	0.01423632
LRRK1	-0.1527975	1.87156709	-3.5503395	0.01433869
TMEM216	0.33888879	2.86652606	3.54876784	0.01436405
GRIK3	-1.4544763	-1.2659303	-3.5482611	0.01437223
LRP5	-0.3408322	3.38583158	-3.5469297	0.01439377
COQ3	0.35914917	1.71870893	3.5468142	0.01439564
CES3	0.46897761	1.03167933	3.53436622	0.01459878
KIAA1715	-0.3840254	1.39607623	-3.533683	0.01461003
NFS1	0.22442271	2.24383191	3.53342486	0.01461428

COX7C	0.29870189	5.89137277	3.53339386	0.01461479
SLC25A17	0.20662587	2.18458717	3.5307472	0.01465845
PTGER4P2-CDK2AP2P2	1.0093305	-1.5132725	3.52957636	0.01467781
GIGYF2	-0.2929803	2.535547	-3.5287584	0.01469135
TREX1	0.30200667	3.03365906	3.52675906	0.01472451
COL4A4	-0.4277248	-1.1028591	-3.5224631	0.01479605
F10	-1.2329134	-0.0831539	-3.5207438	0.01482479
BAX	0.21939174	3.6667134	3.51755863	0.0148782
CDK2AP2	0.36707208	4.06677895	3.5126183	0.01496147
PEX5	-0.2900918	2.6041744	-3.5105871	0.01499586
CASP9	0.24434005	2.29609053	3.50912625	0.01502065
C9orf16	0.37749287	4.78636198	3.5062805	0.01506907
GRAMD3	0.30040186	3.37931507	3.50623765	0.0150698
KIAA2018	0.1762214	1.80804865	3.50487	0.01509313
KRTCAP2	0.24132219	4.87738739	3.49995659	0.01517729
DDB2	0.07631672	3.31140639	3.49912424	0.0151916
SPTY2D1-AS1	1.97435562	-1.3154073	3.49823325	0.01520693
NGEF	0.70314878	1.69075798	3.49815975	0.0152082
CACNA2D3	-1.1261278	-0.4725498	-3.4940418	0.0152793
WIPF3	-0.3043821	0.85678113	-3.491156	0.01532935
SLCO2B1	-0.8472553	0.82476115	-3.4883531	0.01537814
KLHDC3	0.18561772	3.57993761	3.47817744	0.01555673
SPTBN4	-0.4678586	-0.1208375	-3.475172	0.01560992
DCAF13	0.35088994	2.5834803	3.47428702	0.01562562
NUTF2	0.33068832	4.36622447	3.47317192	0.01564543
LOC100287015	0.42894256	0.84885836	3.47263364	0.01565501
EDEM2	0.24997025	2.48892263	3.47128324	0.01567905
IPO7	-0.2555997	2.91213488	-3.4711236	0.0156819

XKR4	-1.3023327	-1.4055612	-3.4702193	0.01569803
CMTR1	-0.2427494	2.58817168	-3.4698023	0.01570547
ZNF174	-0.1729935	1.90189631	-3.4657477	0.01577805
PCDHB19P	-0.6994591	-1.4957002	-3.459974	0.01588205
EIF4EBP2	-0.2248299	3.22053906	-3.4598496	0.0158843
NDUFB11	0.1937032	4.01725886	3.45645474	0.01594582
RYR1	-0.3828021	0.68795779	-3.4552399	0.0159679
PPDPF	0.20369306	6.51647194	3.45271746	0.01601386
TET3	0.21409731	1.61037761	3.44782893	0.01610334
FOXN3	-0.2822071	2.98857281	-3.4474564	0.01611018
FKBP3	0.20148087	3.38067433	3.44664808	0.01612504
PPP1R37	0.13876558	2.93895148	3.43914116	0.01626376
ZYG11A	0.99338026	-1.4631021	3.4302295	0.01643017
CROCCP2	0.28702804	3.37542349	3.42829679	0.01646651
ATXN1L	-0.143217	2.19602246	-3.4240933	0.01654586
LOC101928796	-1.577229	-0.9335611	-3.4228968	0.01656853
MZT2A	0.35154955	3.73680582	3.41623899	0.01669529
MGAM2	-0.5985865	-1.8605681	-3.4150819	0.01671743
ANO5	-0.9502032	-1.1739788	-3.4113867	0.01678835
BBC3	0.51342996	2.0254718	3.41075107	0.01680059
VMAC	0.26373753	2.47385584	3.41008796	0.01681336
GABRB3	0.8539757	0.91506959	3.4100545	0.016814
CTDSP2	-0.2309661	3.70750458	-3.4090541	0.0168333
ZNF791	-0.3142085	2.34031541	-3.4070439	0.01687214
NAP1L2	-0.6279447	1.11852453	-3.4044627	0.01692216
BRI3	0.28628252	4.65210712	3.40249045	0.01696049
HOXB-AS1	1.22627325	0.82308881	3.40244074	0.01696146
EFEMP1	-0.56649	4.15949219	-3.4017718	0.01697448

FAM83E	0.24954992	3.70039955	3.39646623	0.01707818
CALCOCO2	-0.41333	3.11125402	-3.3952849	0.01710137
PDCD1LG2	-1.5511808	-0.6004564	-3.3952001	0.01710303
VPS51	-0.1140878	3.90562841	-3.393215	0.01714208
COX6C	0.27249585	4.68906078	3.39185023	0.01716898
C7	-1.9059428	0.88825052	-3.3899949	0.01720562
MBD5	-0.3564197	0.66706138	-3.3891871	0.01722161
AP4E1	0.31540368	0.96100317	3.38672433	0.01727044
ZBED6CL	0.21657225	1.41815374	3.38643346	0.01727622
YPEL3	0.28749735	4.56194398	3.38110825	0.01738237
PFDN1	0.15229391	3.55591007	3.37840811	0.01743648
RALGDS	0.25009077	3.62466717	3.37524335	0.01750013
H3F3AP4	0.22831306	5.69141175	3.37488844	0.01750728
CIZ1	-0.2469219	3.43361752	-3.3748677	0.0175077
PTCH1	-0.3749406	1.32173284	-3.3727371	0.01755072
GPR143	0.59763958	0.56354829	3.37215882	0.01756242
ARL6IP4	0.16316006	4.72675367	3.36983572	0.0176095
HLA-DQA1	-0.5507164	2.78874923	-3.3698023	0.01761018
RPS13	0.12388138	6.41283703	3.36931244	0.01762012
SH3RF3-AS1	-0.9188657	-1.5146459	-3.3655958	0.01769578
LOC101928414	1.41798535	0.04788611	3.36481032	0.01771182
FAM90A25P	0.49051129	-0.3460038	3.36446709	0.01771883
NDUFS6	0.30622742	5.15958814	3.36245835	0.01775994
SMC1A	-0.3245813	2.0574969	-3.3617972	0.01777349
TRIM59	0.31114066	0.92582306	3.35742015	0.01786351
IL1R1	-0.5247613	3.18285186	-3.3554002	0.01790522
SPATA9	-0.7887847	-1.2133075	-3.3540438	0.01793329
PGM1	-0.2936809	3.06755273	-3.3449427	0.01812291

LAMTOR5	0.34067337	4.18755358	3.34112739	0.01820307
STARD4	-0.2408887	0.71112585	-3.3400448	0.01822589
SEMA5A	-0.5800214	2.34398945	-3.3384157	0.01826028
ANAPC15	0.45175123	3.75658479	3.33290307	0.01837721
LOC441204	-1.5827519	-0.8416016	-3.3326966	0.01838161
PTGES2-AS1	0.85638511	-0.2880283	3.33262176	0.0183832
PUSL1	0.38792742	2.74556208	3.32743606	0.018494
NUP205	-0.1850478	2.12244855	-3.3264756	0.0185146
CLCN7	0.21481436	2.92329633	3.32121062	0.018628
PTGIR	-1.8819902	-0.701041	-3.316659	0.01872666
ANKFY1	-0.1669485	2.41480629	-3.314643	0.01877054
PLEKHJ1	0.45730377	3.28222344	3.30521549	0.01897728
MRTO4	0.15308482	2.71975978	3.30300836	0.01902605
USP25	-0.1477232	2.15203484	-3.3029216	0.01902797
CHMP1A	0.24915587	3.82327824	3.30108216	0.01906873
SPEN	-0.2231555	2.51561027	-3.2999969	0.01909282
LRP12	-0.4088868	1.17329303	-3.2961888	0.01917762
COL13A1	-1.7781826	0.32811212	-3.294943	0.01920546
LDOC1L	-0.1788281	2.22220528	-3.2933797	0.01924045
SRSF6	0.24222315	4.16457218	3.29284199	0.0192525
PPIC	0.30299074	3.45987204	3.29275487	0.01925445
BANF1	0.19340118	4.87015015	3.28581735	0.01941072
ZNF330	-0.1218457	2.80860618	-3.2852491	0.01942358
FAM19A2	-0.9505182	-1.1479067	-3.2817481	0.01950304
GET4	0.2520071	3.59366847	3.28141116	0.01951071
ATP12A	-0.3212623	3.53784143	-3.2776647	0.01959617
HMG20B	0.26177527	4.37941785	3.27691659	0.01961329
SNORD3C	2.20098869	-1.2020908	3.27614209	0.01963103

SLC16A9	0.25826349	2.37120468	3.27380057	0.01968476
PROK2	1.16680269	-1.2927066	3.2737387	0.01968618
PP7080	0.37691631	3.42267032	3.27242374	0.01971644
BLACAT1	0.65103781	0.78171703	3.27174754	0.01973201
SEC23IP	-0.3447197	1.85325546	-3.2703776	0.01976361
KRT10	0.35763633	3.15788572	3.26962328	0.01978103
POLR2J	0.41657643	3.3526583	3.26920747	0.01979064
SEPN1	-0.2166252	3.74317604	-3.2678742	0.0198215
SAE1	0.26000346	3.25301417	3.26747764	0.01983069
ZNF345	0.20262891	1.26862329	3.26719378	0.01983727
SLC35G1	0.50017718	0.02037037	3.26373624	0.0199176
LOC100131257	0.60061935	-1.6860986	3.26285263	0.01993819
GPR65	-0.3056008	-0.9035444	-3.2624858	0.01994674
C5orf66-AS1	0.53124486	1.33173228	3.26133291	0.01997365
NUPL1	-0.131102	2.27949858	-3.260717	0.01998804
LINC01088	-1.0621834	-0.580418	-3.2596781	0.02001235
FIBP	0.28577072	3.43130208	3.258757	0.02003393
REG4	-1.0058395	-1.7996654	-3.253794	0.02015063
DNAJC9-AS1	0.41118704	0.56342834	3.25347458	0.02015816
PCDH11X	-0.7261858	-1.9394922	-3.2517184	0.02019966
PSMG3	0.39927417	3.85357018	3.2516015	0.02020242
PMF1-BGLAP	-1.2479165	1.14261816	-3.2505063	0.02022835
PF4	2.0869774	-0.7400054	3.24964863	0.02024868
TMEM92-AS1	-0.8868391	-1.8591655	-3.2488943	0.02026658
NDRG3	-0.1372533	2.77373094	-3.2472094	0.02030663
ARMCX5-GPRASP2,GPRASP2	-2.3785161	-0.4290694	-3.2449337	0.02036086
MPV17	0.21735215	3.58534604	3.24334496	0.02039881

THSD4	-0.5869591	2.53296777	-3.2404416	0.02046838
SRRM5	0.44260073	0.42600785	3.24005874	0.02047757
GPBAR1	0.97169784	-1.2220993	3.23916744	0.02049899
ARID4A	-0.323539	1.66113609	-3.2363175	0.02056764
C10orf111	0.63882319	0.94319403	3.23507504	0.02059766
GGCT	0.30392206	3.57078227	3.23435449	0.02061508
ANOS1	-0.6846921	2.6700478	-3.2339034	0.020626
DUS1L	0.19616435	4.01518661	3.22394403	0.0208687
PLSCR5	0.557649	-2.0237606	3.22089481	0.02094365
RANBP2	-0.2346078	2.57643635	-3.2204137	0.0209555
CA14	-0.798571	-1.259066	-3.2195397	0.02097705
EXOC6B	-0.5202721	1.0751095	-3.2179939	0.02101522
CC2D1A	0.21539541	3.28130927	3.21737981	0.02103041
RPL34	0.1194024	5.909377	3.21656678	0.02105053
LOC101927844	0.52400896	-2.0405806	3.21621323	0.02105929
TMEM236	0.55447104	-1.643286	3.21533467	0.02108107
C2orf44	-0.211515	1.10983	-3.2134031	0.02112905
NARF	0.3718121	3.15904958	3.21196575	0.02116483
MIR1307	4.10190302	-0.2516336	3.21161673	0.02117352
RBX1	0.36605092	3.72099406	3.20952175	0.02122582
XKRX	0.56935508	0.89447018	3.20865748	0.02124743
TYW3	-0.335652	2.17552136	-3.2054421	0.02132806
ARHGEF33	0.58607629	0.19834496	3.20485659	0.02134278
ZBTB10	-0.1815673	1.19855617	-3.2037892	0.02136965
FAM63B	-0.3855844	1.27947601	-3.203416	0.02137905
ARFGAP1	0.24975761	3.32803868	3.20299369	0.02138969
PDK4	-0.5030451	2.54359498	-3.2008495	0.02144382
OR52N4	-1.6654419	-1.3375541	-3.1990116	0.02149034

CYP3A5	0.71460401	0.12669313	3.19546591	0.02158041
LINC01351	1.01560231	-1.7947839	3.19540755	0.0215819
ZNF426	-0.666971	0.97661061	-3.1938581	0.02162139
NEO1	-0.2125905	2.48256864	-3.1926839	0.02165137
PLXNA2	-0.2739404	2.41837911	-3.1908542	0.02169818
LOC102723344	-0.4823378	0.24366687	-3.1846026	0.02185898
SEMA4G	0.67328462	0.67245394	3.18398895	0.02187483
CWF19L2	-0.2862788	1.88538613	-3.178909	0.02200657
PCF11	-0.2661701	2.52516969	-3.1770999	0.0220537
SETD4	0.25756017	2.31683252	3.17668819	0.02206444
ARID1A	-0.178234	2.77188491	-3.1758115	0.02208733
YAP1	-0.294119	3.53017296	-3.1734829	0.02214826
GOLGA8S	0.54570201	-1.3930961	3.17320333	0.02215559
ATRN	-0.4405798	2.60218917	-3.1687086	0.02227377
SRGAP2	-0.1401767	1.99633242	-3.1683777	0.0222825
GLT1D1	0.83861912	-1.5519701	3.16340916	0.02241401
FLRT3	-0.7033275	2.66404358	-3.1603202	0.02249621
SIGMAR1	0.24241142	3.54821395	3.15562316	0.02262184
DUSP28	0.50615687	1.47049036	3.15551601	0.02262471
LOC100128531	-0.514373	1.32927292	-3.1550898	0.02263615
EIF2B4	0.08489248	2.77578875	3.15198523	0.02271967
SELK	0.2363299	3.5156431	3.15169679	0.02272745
LOC100130075	-1.4791945	-1.5629879	-3.1500982	0.02277061
RRS1	0.26147963	2.81343439	3.14765882	0.02283664
ZNF579	0.29584545	2.6731392	3.14695248	0.0228558
GLUD1	-0.1349136	3.75459052	-3.1464687	0.02286893
TBC1D25	0.16213629	1.88227946	3.1456254	0.02289184
PPP3CB-AS1	0.21259434	1.38667048	3.14386427	0.02293977

DSCR9	1.31608499	-1.3574648	3.14367504	0.02294493
EIF3A	-0.2209722	3.88719141	-3.1409564	0.02301916
GCHFR	0.29421508	3.81910399	3.14012192	0.023042
LINC00853	2.00266254	-0.8416874	3.13962992	0.02305547
SLC6A15	-0.2878549	1.10463961	-3.1388671	0.02307639
PVRL4	0.40617468	3.20405029	3.13828858	0.02309226
CSNK2B	0.24545932	4.48388684	3.13589431	0.02315808
TBC1D5	-0.4191549	2.48316086	-3.1347555	0.02318946
PCDHGB7	-1.0621985	0.9046447	-3.1343691	0.02320012
UBE2V1	0.14074434	4.28342721	3.1335604	0.02322245
PQLC2L	-1.2143223	-1.5984924	-3.132935	0.02323973
GPR55	-0.9140632	-1.2753792	-3.13232	0.02325674
MSC-AS1	-1.237951	-1.1612261	-3.1297318	0.02332847
BABAM1	0.26456246	3.58613565	3.12882435	0.02335367
IRF3	0.31835043	3.77454457	3.12873924	0.02335604
PRR4	-2.7020076	6.88364653	-3.126964	0.02340544
WDR43	-0.2393143	2.42214545	-3.1239204	0.02349041
ZNF331	-0.420353	2.01888021	-3.121048	0.02357092
STARD7	-0.2171096	4.14492986	-3.1204748	0.02358702
GOLGA2P7	0.41800319	2.91339807	3.12017735	0.02359538
KDM3B	-0.1991716	3.10298591	-3.1182414	0.02364988
ZFAND2A	0.26635721	2.22836839	3.11737123	0.02367442
DOCK10	-0.6556577	-0.1724122	-3.1156904	0.0237219
CNIH4	0.34258749	2.84575083	3.11502397	0.02374075
LGR6	-0.5809623	2.66247526	-3.1140902	0.0237672
LINC00958	0.25854231	1.72444415	3.11171168	0.02383471
ADAM12	1.59601149	-0.7056538	3.1096629	0.02389304
ACOX2	-0.2744275	1.24643809	-3.1075788	0.02395253

FEN1	0.32284998	1.82971015	3.10708774	0.02396657
PTRHD1	0.30223987	4.23503856	3.10385618	0.0240592
NAPSA	0.8154258	-0.656354	3.10105551	0.02413981
CLLU1	0.7004129	-1.9523786	3.1006652	0.02415106
CLEC4F	-0.7974147	-0.1474074	-3.0997879	0.02417638
HEATR6	-0.2796011	1.46988936	-3.0981573	0.02422353
SLC15A3	-0.8578515	1.96147557	-3.0980919	0.02422542
KLF15	-0.6123122	1.90408997	-3.0977815	0.02423441
OGFRL1	-0.4471559	2.33730043	-3.0961377	0.02428206
H2AFY2	0.09349353	2.43345153	3.09444132	0.02433135
LOC101929767	0.17109706	2.23598489	3.09435607	0.02433383
TRIM35	-0.3405174	2.1729428	-3.0929186	0.02437568
VPS25	0.27263401	4.14076528	3.08814268	0.02451532
ZRSR2	0.19007174	2.75576176	3.08775792	0.02452661
PAFAH1B3	0.23358598	2.82158212	3.08761813	0.02453071
MRPL22	0.3213912	1.94051	3.0854486	0.02459448
FAM155B	-0.3902603	0.1921147	-3.0850528	0.02460613
TMEM242	0.20588133	1.3844538	3.0785588	0.0247982
CCDC136	-0.4058788	0.87996321	-3.0774523	0.0248311
BARHL1	0.39880488	-2.1031827	3.07622584	0.02486761
ARHGAP19	-0.4008186	0.87233486	-3.0755239	0.02488854
SYNRG	-0.1001728	1.91198141	-3.0750056	0.024904
LOC101927211	1.38903632	-1.6080669	3.0745011	0.02491906
ADAMTS14	0.60316528	1.06202173	3.07374497	0.02494166
LAMTOR4	0.25003307	4.84533057	3.07115096	0.02501934
CLCNKB	-0.5626642	1.12113616	-3.071021	0.02502324
CABYR	0.59846427	1.63504196	3.07079689	0.02502996
ALKBH3-AS1	1.14082785	-1.7321712	3.06804369	0.02511274

ADGRB3	-1.1115619	-1.0411437	-3.0665646	0.02515734
NLGN4Y-AS1	1.33047971	-1.6373452	3.06524544	0.02519719
RPS26	0.25287002	4.95379187	3.06381614	0.02524044
KIAA1217	-0.209417	3.40125616	-3.0619926	0.02529575
RNF115	-0.3305293	2.72081182	-3.061056	0.0253242
UBXN6	0.16382251	4.35451812	3.05908516	0.0253842
SSTR5-AS1	-1.4164078	-0.8999289	-3.057298	0.02543875
ATP5I	0.40036407	5.79819443	3.05670549	0.02545686
RPIA	0.15097026	2.54067556	3.0562583	0.02547053
SDE2	-0.1669007	1.79206251	-3.0558345	0.02548351
KIAA2022	-0.6574826	-0.4527067	-3.05559	0.02549099
UTP14C	-0.2883769	2.23605876	-3.0551546	0.02550433
C15orf54	-0.3271586	-2.1390058	-3.0548466	0.02551377
COX6B1	0.22727825	5.72493385	3.05484652	0.02551377
IKZF3	-0.5713203	-0.2930029	-3.0546951	0.02551841
HUNK	-0.1557062	0.39856054	-3.0544947	0.02552456
RPS8	0.10029945	7.36495381	3.05286233	0.02557468
HSPA13	-0.3311959	1.11572422	-3.0502279	0.02565579
BCLAF1	-0.0825484	3.14053013	-3.0497066	0.02567187
OST4	0.15952461	5.49219733	3.04573705	0.02579471
DBNDD2	0.15653922	3.59878007	3.0443688	0.02583721
ST7L	-0.1940531	1.75996877	-3.0406452	0.02595324
TBL2	0.12765912	2.43731457	3.03879841	0.026011
TMEM215	-0.5627327	-2.0212188	-3.0352535	0.02612228
ATP7A	-0.3632334	1.03391661	-3.0286575	0.02633073
LOC200772	1.37924901	-0.2781865	3.02836866	0.0263399
SEPT7P9	1.72956919	-0.8026639	3.02634132	0.02640436
PGP	0.56016382	2.00017985	3.02582238	0.02642089

TEX264	0.24655739	4.02168177	3.02504644	0.02644562
TREM1	1.48531648	-1.4927109	3.02386954	0.02648319
LOC100287225	0.16074459	-2.2222128	3.02201392	0.02654254
EIF2AK4	-0.3962054	2.31081763	-3.0217256	0.02655177
F2RL3	-1.5816263	-0.5762414	-3.020885	0.02657871
ENDOV	0.30479763	2.05102679	3.01959315	0.02662018
EDNRA	-0.618559	1.55839183	-3.0189241	0.02664169
ADH1A	0.75418202	-1.2530485	3.01819841	0.02666503
LEMD3	-0.3454535	2.00460606	-3.0173101	0.02669364
ATOH8	-0.5868227	1.31415801	-3.0168976	0.02670694
MAX	0.11009537	3.7319232	3.01448522	0.02678484
EXTL2	0.2049401	1.9604651	3.01380856	0.02680674
NASP	0.18551357	3.76607119	3.01037285	0.02691823
ERCC6L2	-0.3468439	1.08467024	-3.0095376	0.02694541
THOC1	0.44000372	2.37319223	3.00817491	0.02698982
MRPL12	0.25297934	3.99928721	3.00785107	0.02700039
SMIM10L2A	-0.8631546	0.20079349	-3.0065459	0.02704302
KRT8P41	0.94368556	-1.6159551	3.00587445	0.02706498
LOC101927746	-2.2355404	-0.1618304	-3.0050375	0.02709238
BAZ1B	-0.1890943	2.70628671	-3.0049311	0.02709586
TMEM92	0.3673272	0.91520595	3.00432613	0.02711569
NCOR2	-0.3078312	3.5156581	-3.0036793	0.02713691
DYNC1H1	-0.1691952	3.31132008	-3.0014444	0.02721036
ZNF585B	-0.1719373	1.53455068	-3.0009962	0.02722511
OR6W1P	0.63251478	-1.9863277	3.00059581	0.0272383
GLG1	-0.174216	2.95669157	-2.9976053	0.02733704
ERN2	0.28420167	3.74916255	2.99723163	0.02734941
CDCA8	0.72509964	0.14345716	2.99712718	0.02735286

ANO3	-0.5509138	-1.5514479	-2.9968104	0.02736335
ZNF296	0.30577451	1.85750034	2.99326383	0.02748109
HAPLN1	-0.8634165	-1.1156955	-2.9899815	0.02759055
ADARB2	-0.6193246	0.73955352	-2.9883252	0.02764596
MC5R	-0.8019658	-1.7958688	-2.9874722	0.02767455
FOXO3	-0.3272172	2.5247443	-2.9865346	0.02770601
PPARGC1A	-0.4337761	1.79316114	-2.9853143	0.02774702
NPM2	0.34378392	2.64372505	2.98204657	0.02785716
MTMR4	-0.1238503	2.18917764	-2.9816725	0.0278698
C17orf49	0.17967021	3.96046863	2.97885177	0.0279653
FAR1	0.22521101	2.30908905	2.97871442	0.02796996
SERPINI2	0.58594435	1.68196728	2.9774292	0.0280136
MFSD10	0.09487976	3.68772211	2.97627028	0.02805302
CNTN4	-1.4224918	-0.1372956	-2.976266	0.02805317
SUSD3	-0.9406781	0.91847783	-2.9716044	0.02821234
NPIPB6	1.09522105	0.14737068	2.96950461	0.02828437
SERPINB6	0.25987323	4.72302729	2.96890543	0.02830496
CDKN2A	1.13001864	2.55436961	2.96832877	0.02832479
EHBP1	-0.2092191	2.32359655	-2.9656537	0.02841699
SNRNP35	0.43614533	3.45816776	2.96455738	0.02845487
TMEM170B	-0.4057969	-0.4813186	-2.9643632	0.02846158
FAM107A	-1.0172375	3.08376294	-2.9637337	0.02848336
DCTN1-AS1	1.25744416	-0.7791272	2.96252295	0.02852531
BACE1-AS	-0.5548414	1.23954466	-2.9622432	0.02853501
TOMM6	0.26352219	4.44414596	2.95350061	0.02884003
WDR73	0.34577949	2.83924192	2.95343461	0.02884234
CDC123	0.15344438	3.58349773	2.95332106	0.02884633
ULK3	0.27931234	3.08723158	2.95126756	0.02891851

WBP1L	-0.2592425	3.45882292	-2.9503876	0.0289495
CNTNAP3B	0.39662285	0.5345954	2.9495822	0.0289779
HDHD3	0.26390949	3.33898455	2.94780056	0.02904083
TUBGCP3	-0.1542162	1.89528367	-2.9472727	0.0290595
MGAT3	-0.5849632	2.34797029	-2.9437683	0.0291838
SNAPIN	0.24256246	3.55891201	2.94197002	0.02924782
RBMS3-AS3	-1.4292644	-0.4385157	-2.9417753	0.02925476
ZFPM2	-1.0671509	-0.5661659	-2.939674	0.02932977
ZNHIT1	0.17505034	3.94575973	2.93847961	0.02937251
IL17RC	0.2289171	3.00884684	2.93387677	0.02953783
LINC00871	0.8012132	-1.9019785	2.93350649	0.02955117
AFF3	-0.4879803	-1.0624446	-2.9331221	0.02956503
ZBTB16	-2.4036567	1.22067308	-2.9327044	0.0295801
LOC100505625	-0.7536563	-1.6537435	-2.9315811	0.02962067
SLC25A44	0.28618023	2.26574265	2.93035666	0.02966496
STYK1	0.4358791	1.5656633	2.93026566	0.02966825
ZBTB38	-0.3733459	2.25322944	-2.9280972	0.02974687
LOC101929372	0.57433868	-2.0154158	2.92559346	0.02983793
TGFB2	-0.5869098	1.4052428	-2.9248522	0.02986495
PSMD9	0.16341585	2.99080756	2.92145776	0.02998902
FEZF2	-0.3383	-2.1334351	-2.9162209	0.03018155
ADAR	-0.2729316	3.5920741	-2.913807	0.03027076
MEN1	0.19689861	3.04166292	2.91292854	0.03030329
MTL5	0.28050909	0.89357672	2.91263558	0.03031415
KLF10	-0.3526425	3.01544113	-2.9118917	0.03034174
BMI1	-0.4070149	2.28944355	-2.9092105	0.03044141
AP2S1	0.44444321	3.75206803	2.90798333	0.03048715
GRID1	-1.1125339	-0.9540743	-2.9056934	0.03057271

RPS6KB2	0.13418271	3.35184712	2.90326937	0.03066356
LINC00479	0.39667535	0.90244715	2.90282088	0.0306804
TOX3	-0.5801316	2.20925929	-2.9015875	0.03072677
ZNF544	0.1717228	2.26830229	2.89937244	0.03081024
MYCBP2	-0.3228176	1.59348632	-2.8978502	0.03086775
LINC00968	-1.3737492	-1.1874368	-2.8969843	0.03090051
ECI1	0.24260485	4.00703369	2.89693968	0.0309022
FAM19A1	1.10195533	-1.7516074	2.89409441	0.03101014
HLCS	-0.2732356	1.8134401	-2.8932854	0.0310409
FBXL6	0.4805716	2.18948037	2.89197536	0.03109079
MUC5B	-1.3116853	4.45928352	-2.8916968	0.03110142
LINC00862	0.78101449	-1.9120778	2.89164411	0.03110342
NUCKS1	-0.2399809	3.90769559	-2.8911835	0.03112099
SUCNR1	-1.3693858	-1.1426383	-2.8893135	0.03119243
LINC01343	-0.5898682	-1.9447138	-2.8892398	0.03119526
TMEM105	-0.6501054	-0.4338976	-2.8862784	0.03130878
MSH6	-0.1709732	2.54509744	-2.8862397	0.03131026
DPY19L4	-0.3181845	1.79321398	-2.8861921	0.03131209
LOC113230	0.41656706	2.54612393	2.88580646	0.03132691
GSS	0.22671446	3.25187198	2.88471005	0.03136908
BRF1	0.18057049	3.07535013	2.88435114	0.0313829
NCKAP1L	-0.6636931	0.18069581	-2.8843163	0.03138424
LDAH	0.16745471	1.47559069	2.88364553	0.03141008
SPECC1L	-0.1619979	2.52280724	-2.881914	0.0314769
SOS2	-0.4598003	2.01944544	-2.880137	0.03154564
GEMIN8P4	0.70527501	0.54409288	2.88009986	0.03154708
CCDC7	0.83876884	0.10068629	2.87897493	0.03159069
EIF4B	-0.2246611	4.92702281	-2.8772703	0.03165688

NARFL	0.32422246	2.81788054	2.87663199	0.03168171
NUP85	0.21970265	2.36146885	2.8764165	0.0316901
WDR45	0.1912152	3.29095411	2.87624025	0.03169696
CACHD1	-0.1465126	2.80383907	-2.8759943	0.03170654
PTPRG	-0.7678034	1.10134303	-2.8754976	0.03172589
ZNF536	-1.0720201	-1.7160042	-2.8729655	0.03182474
TOX	-1.1108929	-0.2279364	-2.8729308	0.03182609
KIF13A	-0.3066279	2.46458893	-2.8728875	0.03182779
SRGAP2C	-0.9514869	-0.5081526	-2.87194	0.03186487
DUOXA2	1.51910421	-0.9014756	2.87171275	0.03187377
FKBP1A	0.17943514	4.61687881	2.87126945	0.03189114
GFRA1	-1.3828065	0.69847278	-2.8711328	0.03189649
LOC729966	0.77365022	0.97097221	2.86933706	0.03196697
NADK	0.14154434	3.31051	2.86653571	0.03207727
PJA2	-0.3673254	3.05565818	-2.8662794	0.03208738
PDGFC	-0.5296586	2.04044917	-2.8661424	0.03209279
ARHGAP9	0.26127886	1.01107594	2.86590434	0.03210218
LOC283575	0.83375665	-0.9166635	2.86484182	0.03214416
FGF2	-1.1896454	-0.3071948	-2.8646038	0.03215357
KLHL9	-0.1860177	2.59911201	-2.8644968	0.0321578
LOC101927587	1.03052224	-1.5183097	2.86357358	0.03219434
PRPF4B	-0.1604956	2.58558378	-2.8635224	0.03219637
ALDH2	-0.1214167	5.17903997	-2.8633886	0.03220167
PACERR	1.79624548	-1.4044624	2.8633876	0.03220171
TSHZ2	-0.5354211	1.97904166	-2.8625364	0.03223545
EPS8	-0.2325675	3.28841704	-2.861054	0.0322943
SLC1A5	-0.3392684	4.85557097	-2.8609901	0.03229684
SLX4IP	0.50658952	0.88698779	2.86082924	0.03230323

KCNA3	-0.4770351	-0.9787323	-2.8600194	0.03233544
MAP4K1	-0.5601995	0.42986667	-2.8585866	0.03239251
KPTN	0.26973518	1.46003836	2.85837979	0.03240076
GGN	1.13793267	-1.0616128	2.85801256	0.03241541
SCAF11	-0.1287995	2.90863034	-2.8571735	0.03244891
PPARA	-0.2448449	1.3032043	-2.8558341	0.03250246
ROCK1	-0.2592861	2.47607749	-2.8540379	0.03257443
C5orf66	0.27407999	1.3913406	2.85323562	0.03260663
EP300	-0.1372969	2.7011937	-2.8524975	0.03263629
LINC01002	0.58087118	-0.5361875	2.85191219	0.03265983
EDF1	0.18040459	5.91023196	2.84947475	0.03275805
METTL17	0.40010025	3.09853045	2.84836486	0.03280289
SNORA8	3.54104703	-0.5320616	2.84557392	0.03291592
NR2E3	1.34693545	-0.812317	2.84364284	0.03299438
CRISP3	-1.8891357	1.79912533	-2.8425256	0.03303987
C9orf89	0.38258663	3.11137781	2.84246202	0.03304246
C18orf21	0.32182687	2.59166843	2.84208642	0.03305777
ADGRE3	0.77491227	-1.3396959	2.84142745	0.03308465
CACNG2	0.52545265	-2.0398588	2.84130254	0.03308975
TDG	0.28028303	2.05496837	2.84084572	0.0331084
QRICH2	0.34570561	1.15612881	2.84028524	0.03313129
UBE2W	-0.1184912	1.26051546	-2.8389018	0.03318789
LOC339593	0.69883445	-1.9531679	2.83565131	0.03332127
BCAM	-0.5230603	5.5887703	-2.8353726	0.03333273
TEKT4P2	0.62707036	1.20303696	2.83349717	0.03340999
DGUOK-AS1	0.59168588	2.02357103	2.83249937	0.03345117
KCNE5	1.45567837	-1.5747459	2.83239134	0.03345563
PIK3R1	-0.6850633	3.00365513	-2.8319289	0.03347474

TMTC1	-0.6493867	1.36342152	-2.8309864	0.03351372
RBM48	-0.1353452	2.18134694	-2.8309415	0.03351558
MAN1A2	-0.3232369	2.05955033	-2.8305231	0.0335329
MTHFSD	0.31023383	2.32933122	2.83032159	0.03354125
SLC39A10	-0.2484741	1.39999427	-2.8302091	0.03354591
CCDC18	0.5420589	0.04831382	2.8301529	0.03354824
JTB	0.20750288	4.28198703	2.82731026	0.03366624
GPS1	0.14198211	3.94043996	2.82718574	0.03367142
CMC2	0.6736018	2.64257468	2.82560389	0.03373729
IGF2BP2	0.54232381	2.7881392	2.82405579	0.0338019
LOC101928489	0.62372066	-0.7741485	2.82403226	0.03380288
ZC3H12B	-0.7280651	-1.3628857	-2.8237291	0.03381555
KCNC1	0.28706145	-1.6820049	2.82193264	0.03389072
ADGRG3	0.76560562	-0.9058145	2.82055549	0.03394847
CHMP4B	0.20294692	4.86574991	2.81979339	0.03398047
KREMEN1	-0.1680293	3.05753639	-2.8197107	0.03398395
MRPL15	0.17269419	2.83133371	2.81950946	0.0339924
NOP10	0.45403028	4.52427376	2.81936769	0.03399836
IPO5P1	0.18134325	1.67126139	2.81840733	0.03403877
GBP4	-0.4900546	1.58544843	-2.8173526	0.03408319
GXYLT2	-0.5188172	1.5704433	-2.8170243	0.03409704
HLA-DRB5	-0.3880868	4.02436034	-2.8159464	0.03414253
CCDC57	0.37918605	3.05135394	2.81580749	0.0341484
ZNF738	0.34206802	0.1806038	2.81489731	0.03418687
UGGT1	-0.1927906	1.92129242	-2.8132744	0.03425559
BTBD8	-1.3079256	-0.4699087	-2.8129753	0.03426827
OTOGL	-0.4156478	-2.0037199	-2.8120879	0.03430593
PLA2G4C	-1.8326482	-0.178378	-2.8114946	0.03433113

CNTFR	-1.8142948	-0.6194369	-2.8112421	0.03434186
ZBTB44	-0.1192152	2.4486419	-2.8105139	0.03437282
DLGAP1-AS2	0.84777985	0.64944941	2.80903672	0.03443574
RCSD1	-0.6939174	0.6330811	-2.807353	0.0345076
TMSB4Y	0.7345737	0.98175115	2.80732539	0.03450878
BPIFB1	-0.3518731	7.90950614	-2.806909	0.03452658
ASMTL-AS1	0.4923194	1.86110432	2.80614547	0.03455924
CCDC159	0.4064899	2.67466601	2.80464765	0.03462342
FAH	0.2490757	1.89045341	2.80372651	0.03466295
WDR27	0.31376985	1.987808	2.79865981	0.03488126
MRPL33	0.14268315	4.7815724	2.79837384	0.03489363
ZFPM2-AS1	1.08493654	-1.6802321	2.79827708	0.03489781
ATP5E	0.30891689	6.04836603	2.79535824	0.03502433
ZNF462	-0.2516191	1.7986123	-2.7898607	0.03526398
GRHPR	0.09780862	4.06285716	2.78930357	0.03528837
RNFT2	0.51172114	0.80152943	2.78784819	0.03535216
FAM173B	0.18318019	1.5655661	2.78683255	0.03539675
TMEM100	-1.9694568	-0.3387052	-2.7850372	0.03547573
HERC1	-0.2143203	1.54089511	-2.7846909	0.03549098
SEC24C	-0.1626455	3.48057433	-2.7840081	0.03552108
LINC00116	0.28895724	2.20792425	2.78307804	0.03556213
SGCB	-0.2956924	2.4350206	-2.7809311	0.03565707
ADPRHL2	0.44417926	3.60942278	2.77835019	0.03577158
FCGR3B	1.37094168	-0.347098	2.7771202	0.03582629
SUV420H2	0.39013857	2.39548241	2.7759979	0.03587629
RPL3	-0.1811851	7.03435986	-2.7751545	0.03591392
EXOSC6	0.28883116	3.19895231	2.77333031	0.03599544
GALNT18	-0.221507	2.72837946	-2.7730426	0.03600832

BTLA	0.81402517	-1.4071094	2.77299486	0.03601046
SF3A1	-0.3507328	3.38304122	-2.7685141	0.03621166
CD99L2	-0.1414061	2.92285556	-2.7683683	0.03621823
CTD-3080P12.3	0.6010356	-1.5689363	2.76654188	0.03630061
2-Mar	0.2253978	3.2099474	2.76438969	0.03639795
SMIM12	0.23658558	1.65265397	2.76309507	0.03645664
RASSF6	-0.3884292	3.02493961	-2.7623136	0.03649212
MAEA	0.09407241	3.6076823	2.76206121	0.03650358
IL27RA	-0.4991006	1.78362489	-2.7614969	0.03652923
KCP	1.32970808	-1.4592561	2.76051456	0.03657393
VPS4A	-0.1398525	3.68159273	-2.7553805	0.03680852
NDUFC1	0.24684409	4.4436609	2.75477959	0.03683608
F13A1	-0.8971998	0.4056216	-2.7542644	0.03685973
CHPT1	-0.3819217	2.87676668	-2.7536057	0.03688999
NOC2L	0.13819293	3.40777701	2.75324368	0.03690664
SFT2D3	0.18994608	2.37223991	2.75125662	0.03699814
CACNA2D2	-0.5250234	-0.1118787	-2.7503679	0.03703914
GYS1	0.17790938	2.69288884	2.74984829	0.03706313
GJC2	-0.8020599	1.65496724	-2.7493578	0.0370858
AMH	1.32856729	-0.4517036	2.74887055	0.03710833
PQLC2	0.2517412	2.09524016	2.74747853	0.03717279
INVS	-0.2925265	1.23511944	-2.7470205	0.03719402
PAPOLG	-0.1706029	1.09387854	-2.7459293	0.03724467
TFAP2B	0.41049799	-1.4498969	2.74417097	0.03732643
GPX1	0.36528659	5.79272294	2.7432721	0.0373683
NDUFA7	0.30179937	4.4755039	2.74207334	0.03742422
ARNT	-0.1021883	2.60655869	-2.7413846	0.03745639
LOC100129175	0.66340801	0.13330749	2.7393204	0.03755299

MICU3	-0.6945271	0.05767022	-2.7383922	0.03759652
CHRM1	-1.7178017	-0.6298528	-2.7371941	0.03765278
CFLAR	0.12367256	3.63601208	2.73692018	0.03766566
CCDC149	-0.3121316	1.75017373	-2.733421	0.03783058
PFDN2	0.31557012	4.05443622	2.73205713	0.03789506
SIGLEC6	-0.9024625	-1.8513538	-2.7318209	0.03790625
VSIG4	-1.5523347	-0.2915148	-2.7316898	0.03791245
ATP11B	-0.2545801	2.3357568	-2.7311003	0.03794038
MLPH	0.43410411	3.52203754	2.7285176	0.03806299
TMEM175	0.18037914	3.39082014	2.72659657	0.03815447
NUP160	-0.2345376	2.07860069	-2.7257568	0.03819453
SGTB	-0.5481932	-0.0304783	-2.7254397	0.03820967
ATAD3A	0.19548413	2.97405868	2.72499181	0.03823107
LIMCH1	-0.5997429	2.52354505	-2.7220843	0.03837028
GNPTG	0.14537941	3.99919611	2.72192195	0.03837808
C3orf14	0.29559607	0.80741429	2.72156077	0.03839541
ZNF341-AS1	1.22962699	-1.4758393	2.72137761	0.03840421
BBX	-0.2849518	2.04489113	-2.7208777	0.03842822
GPHN	-0.3147932	1.2363198	-2.7206412	0.03843959
LOC643072	0.32243189	1.40249008	2.72063271	0.03844
CTDSPL2	-0.205314	1.53747552	-2.7198481	0.03847773
TSPAN17	0.17814158	2.86841175	2.71955429	0.03849187
RNF149	0.15227463	2.80426634	2.71820252	0.03855701
KIAA2013	0.11417474	3.3846158	2.7180341	0.03856513
RFC1	-0.3181879	2.25193455	-2.7175195	0.03858997
MBTPS2	-0.175825	1.25830934	-2.7162736	0.03865016
ALDH7A1	-0.1075253	2.60305093	-2.7155353	0.03868589
FAM26E	-1.3413992	-0.4221747	-2.7146389	0.0387293

NUP98	-0.1808351	2.61595343	-2.7121371	0.03885076
PDE6D	0.59269637	2.83437805	2.71177484	0.03886838
ZCCHC11	-0.1501908	2.05608912	-2.7113594	0.0388886
SPPL2B	0.30877534	3.48051612	2.71105971	0.03890319
FAM20B	-0.1668262	2.42186413	-2.7108375	0.03891401
ANGPTL1	-0.9364496	0.01371366	-2.7100046	0.03895461
GIPC1	0.37077417	4.46429146	2.70971807	0.03896859
PRNP	-0.5027794	4.16994116	-2.7087749	0.03901464
TADA2A	-0.3193595	1.98546728	-2.7044399	0.03922704
SSBP1	0.18434423	4.54270674	2.70429375	0.03923422
SLC7A8	-0.2237241	2.78653197	-2.7033373	0.03928127
SLC22A16	1.20419078	-0.4415165	2.70200323	0.03934698
ZC3H13	-0.3351053	2.05787039	-2.6992897	0.03948103
ANKRD13D	0.19701082	3.3472295	2.69908971	0.03949092
MIEN1	0.38514193	4.31816676	2.69866534	0.03951194
FAM49A	-0.5319369	0.34233085	-2.6968191	0.0396035
ABHD16B	0.43387881	2.38573842	2.69594976	0.03964669
COX6A1	0.37593153	6.03412912	2.69473973	0.0397069
LOC100505715	1.43882548	-0.6079882	2.69457119	0.03971529
PPM1L	-0.443894	1.47405604	-2.6939104	0.03974822
PPP2R2D	0.26222216	1.92754676	2.69274253	0.03980649
UBR3	-0.1620982	1.75529312	-2.6925856	0.03981432
USP15	0.10833087	2.12456831	2.69036759	0.03992527
COA7	-0.471467	1.20023124	-2.690254	0.03993096
STAC3	0.48057547	0.68101501	2.69022086	0.03993262
KRTAP5-AS1	1.35406489	-0.4756219	2.68993755	0.03994682
TMOD2	-0.6843353	0.68447851	-2.6896716	0.03996015
IMPDH2	-0.19453	4.27900827	-2.6889646	0.03999562

SLC25A29	0.350789	4.19096231	2.68852331	0.04001777
ELAVL3	0.67826732	-1.6772818	2.6878442	0.04005189
PSTPIP1	-1.0888742	0.75076362	-2.6873878	0.04007484
CASS4	-1.4526009	-1.1493864	-2.6873475	0.04007687
ZIC5	0.23109988	-2.1870352	2.68664544	0.0401122
HIGD1B	-1.8624167	-0.2948459	-2.6866147	0.04011375
PCDHA10	-0.7990734	-0.9774025	-2.6860161	0.0401439
COMTD1	0.43664353	2.68187085	2.68595426	0.04014702
KIF20B	-0.2614522	0.30300936	-2.6847295	0.04020879
ASPSCR1	0.26156474	2.90837058	2.68411402	0.04023988
PID1	-0.953058	1.70882493	-2.6827441	0.04030915
SEMA6C	0.46369282	1.32483671	2.68245727	0.04032368
GBP1	-0.3272594	2.31292578	-2.6807278	0.04041136
SEMA3E	-0.5974273	0.23527859	-2.6801589	0.04044024
PELI3	-0.5474493	1.52653238	-2.6765518	0.04062393
GNG10	0.25449176	3.23957758	2.6757736	0.04066367
CDK5RAP2	-0.2573533	2.65220409	-2.6754408	0.04068068
XPO1	0.06293013	3.33419446	2.6753812	0.04068373
DNASE1	0.48400221	0.91590085	2.67500723	0.04070285
LOC101927204	-0.5147714	0.91877091	-2.6749164	0.0407075
NRGN	1.1957281	1.30088719	2.67366246	0.04077171
PRND	1.48914036	-1.3978471	2.67219275	0.04084711
LINC00607	0.63160101	-1.2889156	2.67176628	0.04086901
SPATA1	1.29377721	-1.3114836	2.67125063	0.04089552
PABPC1	-0.3045539	6.16703442	-2.6708307	0.04091712
CDIPT-AS1	1.13835832	-1.1272203	2.67045262	0.04093657
THUMPD3-AS1	0.41229537	1.89436979	2.6696399	0.04097843
ITPKA	0.48379478	0.87691363	2.66950717	0.04098527

TXNIP	-0.4665054	5.74491622	-2.6688843	0.04101739
FLJ37201	0.71590892	-1.3357538	2.66875816	0.0410239
ZNF529	0.38877801	1.78845517	2.66785498	0.04107052
MRPS24	0.3332593	4.4841724	2.66783699	0.04107145
COBLL1	-0.4089571	1.32916353	-2.6677878	0.04107399
RILP	0.23509496	2.12301549	2.66760536	0.04108342
CD79B	-1.2454533	0.80453634	-2.6675955	0.04108393
FTSJ2	0.2772561	2.86248127	2.66744557	0.04109168
PTPRU	-0.1919592	3.97605781	-2.6668747	0.0411212
GNG3	1.22552484	-0.5445593	2.66634484	0.04114861
TYMP	0.24762634	4.63203659	2.66595211	0.04116895
HLA-DRB2	-0.3497721	1.89441846	-2.6631467	0.04131452
TCERG1	0.14986762	2.77948936	2.66197918	0.04137527
OXLD1	0.27552166	3.36030684	2.66131661	0.04140979
MED12	-0.2042218	2.30021018	-2.6609637	0.04142819
FAM106A	1.19796835	-0.9932131	2.65968175	0.0414951
FAM127B	0.16504617	4.03350655	2.65955539	0.0415017
HLA-DRB1	-0.3687934	4.36656982	-2.6589987	0.04153079
RC3H1	0.15774136	1.80534311	2.65864814	0.04154913
GOLGA7	-0.082104	3.26369577	-2.6582993	0.04156738
LOC389705	-0.7353881	-1.3970849	-2.6581448	0.04157546
NTRK3	-1.1044526	-0.5895418	-2.6578513	0.04159083
GIPR	0.69012995	2.96074765	2.65705253	0.04163269
TACR1	-1.5166756	-0.4880046	-2.6568106	0.04164537
NELL1	-0.7068017	-1.8094366	-2.6561233	0.04168143
LOC101928035	0.71320642	-1.9459819	2.65514069	0.04173304
NEAT1	0.59783679	5.99174124	2.65436551	0.04177381
CHST10	-0.3465459	1.62193679	-2.6543442	0.04177493

LOC101929124	0.53826767	-2.0098208	2.65340544	0.04182436
SORCS1	-0.9224317	-1.0283912	-2.6524131	0.04187668
ADH1B	-1.979033	2.06457856	-2.6518764	0.04190501
THAP7-AS1	0.39233098	2.17525336	2.65151413	0.04192414
HNRNPD	0.03139227	4.67791039	2.64956866	0.04202704
CA11	-0.7320263	1.79542678	-2.6490069	0.04205681
FYCO1	-0.314681	2.37732327	-2.647272	0.04214887
RSL1D1	-0.1964623	3.24230833	-2.6469257	0.04216728
PKN2	-0.1674951	2.3497488	-2.6468342	0.04217214
TCF20	-0.1383489	2.5671447	-2.6454239	0.04224719
U2AF2	0.08663438	4.14120242	2.64473223	0.04228405
POLR1D	0.16005007	4.43521991	2.6433497	0.04235782
ACSS3	-0.5265375	2.0322681	-2.6425224	0.04240204
FLNB	-0.1644939	4.22074233	-2.6404305	0.04251407
R3HDM4	0.31980509	3.72304448	2.63694451	0.04270147
FLYWCH2	0.27330703	3.68420042	2.63632027	0.04273512
ZDHHC9	0.13401815	2.07572891	2.63586526	0.04275967
PAFAH1B1	-0.1541357	3.44658333	-2.63552	0.04277831
PPP1R16B	-0.5157697	1.95892122	-2.6349725	0.04280788
RNASEH1	-0.3443585	1.76061563	-2.6334377	0.04289089
ZNF507	-0.1504779	1.7636442	-2.6323587	0.04294936
ZNF721	0.13913424	2.11251846	2.6314543	0.04299843
LOC729737	0.57482073	1.81271862	2.6295814	0.04310024
PGC	0.52909737	-2.0380364	2.62953433	0.0431028
UBAC2-AS1	0.47276637	0.41223558	2.62901931	0.04313085
SSR3	-0.1517548	2.86314155	-2.6287633	0.04314479
ITGB5	-0.1408353	3.53638383	-2.6270955	0.04323578
CKMT2	-1.2741407	-0.5170163	-2.6246311	0.04337061

CP	-0.1805462	4.06811118	-2.6242776	0.04338998
SCAPER	-0.4841774	1.02348433	-2.622557	0.04348443
TBCB	0.24497696	4.38910756	2.62246372	0.04348956
RBMS3	-0.6603396	1.52294652	-2.6223133	0.04349783
LRRC28	0.30192906	1.77430956	2.62165797	0.04353387
YRDC	0.23539515	2.017641	2.62147375	0.04354401
LOC101928710	1.07919791	-1.7629861	2.61878615	0.0436922
LOC101929572,POTEH-AS1	0.49963515	-2.0527675	2.61859917	0.04370253
PABPN1	0.22542261	3.73213479	2.61808139	0.04373115
YBX3	0.17022588	5.04637846	2.61744563	0.04376632
USF1	0.25398878	3.48443759	2.6173446	0.04377191
LOC101929468	-0.6666589	-1.9203249	-2.6168007	0.04380203
KIDINS220	-0.1625149	2.52966826	-2.6158761	0.04385328
KIAA1919	0.44393557	0.6992951	2.61566241	0.04386513
PCK2	-0.2460582	2.34863613	-2.6154952	0.04387441
GAPDHS	1.49819193	-0.8674857	2.61523913	0.04388862
SCAMP3	-0.1773311	3.42174334	-2.6137774	0.04396984
SRL	-0.6427867	-1.0972053	-2.6124407	0.04404426
STK36	0.52199273	2.65255783	2.61230002	0.0440521
RASL10A	1.52386507	-0.7820534	2.61189137	0.04407488
RMND5A	-0.2624423	2.46614118	-2.6108013	0.04413571
HIP1R	0.21371634	2.98148929	2.60986235	0.04418819
CDC73	-0.2145312	1.84363387	-2.6091523	0.04422791
SAMD10	0.23256929	1.92723078	2.60652479	0.04437525
DUSP16	-0.307696	2.09335341	-2.6050685	0.04445714
ITGA8	-1.5578408	0.50160409	-2.6048987	0.0444667
FIBIN	-0.7878679	1.21856853	-2.6029979	0.04457386

SLC14A2	-0.1721499	-2.2165101	-2.6014768	0.04465981
NR2F2	-0.4526841	3.80641599	-2.6014741	0.04465996
SLC27A6	-1.472746	-0.7809653	-2.6014637	0.04466055
TWIST2	-1.4844697	0.85737562	-2.6014503	0.04466131
ALX1	1.14395651	-0.9542732	2.60139216	0.0446646
BANK1	-0.4466454	1.00237505	-2.6012464	0.04467285
LOC105274304	0.45406835	0.33192918	2.60097309	0.04468831
SERF2	0.33404486	4.74501403	2.59754727	0.04488269
HERC2P7	0.56217426	-1.7310066	2.59730529	0.04489645
DRG2	0.28657677	3.02600091	2.59508909	0.04502272
APOOL	-0.358674	1.332612	-2.5941681	0.04507531
ETV4	-0.5899699	1.4183724	-2.5939578	0.04508733
ATP6V1G3	-1.4306892	-0.0345134	-2.5929838	0.04514303
ATRX	-0.2243983	2.05847811	-2.5928662	0.04514976
OLFML2A	-0.1776273	2.4883682	-2.5914138	0.04523297
ZMIZ2	0.22949568	3.39894877	2.59065192	0.04527669
PARM1	-0.3374018	2.37900763	-2.5876734	0.04544804
PAM	-0.4355422	3.2678847	-2.5866424	0.04550752
BPIFB2	-1.7809447	4.86611008	-2.5853887	0.04557996
CCDC9	-0.1967635	2.70214726	-2.5853158	0.04558417
CCDC61	0.27164306	2.79467336	2.58180403	0.04578775
NFYB	-0.2561505	2.40734504	-2.5812071	0.04582246
RAB17	0.23090182	2.28215378	2.5809107	0.0458397
ZNF57	0.27035267	1.85510735	2.58026749	0.04587714
TMEM204	-0.5073519	2.81902491	-2.5789489	0.045954
IP6K1	0.14900871	2.86021966	2.57868048	0.04596966
ZNF273	0.46474107	2.68586089	2.5783936	0.0459864
METTL21EP	0.8284246	-0.9423195	2.57795686	0.04601191

NME1	0.4178265	3.8507493	2.57756112	0.04603503
GPR12	-1.1956905	-1.3176783	-2.5767164	0.04608444
ZCCHC2	0.26672239	1.45742028	2.5760687	0.04612236
CDKN1B	-0.2862145	3.45526918	-2.5756376	0.04614761
NPEPPS	0.12961534	3.28192977	2.57522872	0.04617158
SCYL3	0.30261358	1.9328545	2.57491513	0.04618998
KIAA0040	-0.3229171	2.15904764	-2.574753	0.04619948
SSC4D	1.14125521	-0.8157531	2.5735593	0.04626959
SLC35E2B	0.18370949	2.73514126	2.57241049	0.04633716
PFAS	-0.1751318	1.41998795	-2.5722801	0.04634483
ABCA7	0.30135487	2.79156	2.56885292	0.04654709
LBX2-AS1	0.69025378	0.82325282	2.56849976	0.04656798
HLF	-0.6239737	2.66360947	-2.5663745	0.04669394
MSR1	-0.6951696	-0.3598777	-2.5646391	0.04679706
C10orf76	-0.1948447	1.99362438	-2.562386	0.04693132
LOC100507053	0.34360216	1.28912378	2.55987196	0.04708162
MRPS33	0.33516401	3.80308193	2.55846565	0.04716592
REST	-0.1583146	2.05554212	-2.5583284	0.04717415
TNKS2	-0.1175271	2.7921579	-2.5575327	0.04722194
DCUN1D5	0.17715622	0.68370318	2.55726973	0.04723773
SLC16A14	-0.3693248	1.27768658	-2.5570557	0.0472506
CD177	0.78527979	1.23124496	2.5568218	0.04726466
YBEY	0.27980914	2.80902737	2.55653746	0.04728177
IMMT	-0.1444003	3.12897577	-2.5561191	0.04730694
RRP7A	0.18430212	2.59740785	2.55489783	0.04738052
ZMYND11	-0.308221	3.26440913	-2.5541204	0.04742742
